# Tetracycline-loaded nanomotors target neutrophil activation and oxidative stress in acute lung injury

**DOI:** 10.1016/j.isci.2026.117106

**Published:** 2026-08-06

**Authors:** Saiqi Li, Ziqiang Zhang, Qianyun Zhang, Yi Shen, Chong Zhang, Jia Huang, Fan Su, Yuning Sun

**Affiliations:** 1 School of Health Science and Engineering, University of Shanghai for Science and Technology, Shanghai 200093, P.R. China; 2 Department of Critical Care Medicine and Emergency, Shanghai Chest Hospital, Shanghai Jiao Tong University, Shanghai 200030, P.R. China; 3 National and Local Joint Engineering Research Center of Biomedical Functional Materials, School of Chemistry and Materials Science, Nanjing Normal University, Nanjing 210023, P.R. China; 4 Department of Cardiology, Zhongshan Hospital, Fudan University, Shanghai Institute of Cardiovascular Diseases, Shanghai 200032, China; 5 Department of Pediatric Endocrinology and Genetic Metabolism, Xinhua Hospital, Shanghai Institute of Pediatric Research, School of Medicine, Shanghai Jiao Tong University, Shanghai 200092, China

**Keywords:** acute lung injury, caspase-1 signaling, oxidative stress, nanomotor, nanomedicine

## Abstract

Acute lung injury (ALI) has high mortality, underscoring the urgent need for effective therapies. Excessive inflammation, mainly driven by neutrophil activation and caspase-1-mediated IL-1β/IL-18 release, along with oxidative stress, plays a pivotal role in ALI progression. Tetracycline (TH) has shown promise in suppressing caspase-1-inflammasome signaling, and its therapeutic efficacy could be further enhanced through targeted delivery. Here, we developed a nitric oxide (NO)-driven nanomotor for reactive oxygen species (ROS)-responsive TH delivery (PMA@TH), enabling precise TH delivery to inflammatory lung tissues. Upon intratracheal administration, PMA@TH migrates to injury sites with high ROS concentrations to achieve precise drug release and the generated NO further enhances tissue penetration. The nanomotor not only inhibits neutrophil infiltration but also promotes macrophage M2 polarization, mitigating both local and systemic inflammation. Transcriptomic analysis further confirmed the dual anti-inflammatory and antioxidant effects of PMA@TH. This strategy demonstrates superior therapeutic outcomes in ALI models, offering a promising nanomedicine for clinical translation.

## Highlights

• Inhalable NO-driven nanomotor carries tetracycline for acute lung injury
• Released NO drives nanomotors to high ROS sites, enhancing targeted therapy
• It combines ROS clearance and immune modulation for synergistic lung therapy

## Graphical abstract

**Figure:**
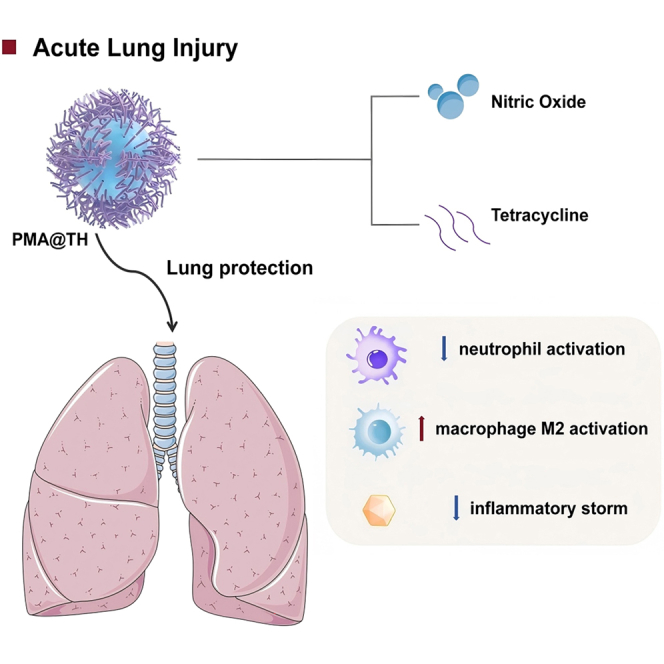

## Teaser

Drug delivery system; Biological sciences; Immunology; Biotechnology; Biomaterials; Nanomaterials

## Introduction

Even with advanced treatments, acute lung injury (ALI) usually causes substantial respiratory failure and mortality, with mortality rates up to around 40%.^1^^,^^2^ Further, ALI survivors usually suffer from long-term disabilities, tremendously reducing the quality of daily life.^3^ Thus, novel therapeutic methods are urgently needed to inhibit the inflammatory cascade to promote the ALI prognosis. Lung inflammation arising from the activation of the innate immune system is the primary characteristic of ALI,^4^^,^^5^ especially the activation of neutrophils.^6^^,^^7^ Accumulating evidence has highlighted the key roles of IL-1β and IL-18 in the development of ALI,^8^^,^^9^^,^^10^ suggesting that the inhibition of the pathways responsible for the production of IL-1β and IL-18 should be beneficial for ALI patients. A series of investigations targeting the production of IL-1β and IL-18 have been performed^11^^,^^12^^,^^13^^,^^14^; in particular, the inflammasome-caspase-1 pathway has attracted increasing attention. Tetracycline (TH) can be used to treat the IL-1β- and IL-18-mediated inflammatory diseases, and recent investigations have proven its ability to block inflammasome-caspase-1 signaling in ALI.^15^^,^^16^ Establishing a precise delivery platform of TH is an effective way to further improve efficacy. Meanwhile, over-activation of the reactive oxygen species (ROS) system represents another important factor in lung damages,^17^^,^^18^ which not only directly causes oxidative stress damage in the lungs but also further promotes the activation of inflammatory cells, worsening the inflammatory cascade.^19^^,^^20^ To conclude, the over-expression of ROS is generally accompanied by over-activated immune cells, which can serve as a potential target for TH delivery.

In recent years, a series of nanomotors have been developed as a means of precise drug delivery, and exciting achievements have been obtained to support their biomedical applications in various diseases.^21^^,^^22^^,^^23^ By sustained release of gases, generally H_2_ or CO_2_, mechanical power is provided and the movement of nanomotors is motivated, so as to realize the drug delivery.^24^^,^^25^^,^^26^ We have successfully established a nitric oxide (NO)-driven nanomotor, the movement of which is effectively triggered by the specific high concentration of ROS at the injured sites, thereby enhancing their precise delivery capabilities to injured sites.^27^^,^^28^ Moreover, different from other nanomotors based on H_2_ or CO_2_, which may be toxic to the human body, the sustained NO release of our nanomotor has been proved to have protective effects on endothelial cells.^28^ The above features suggest that NO-based nanomotors hold the promise to be drug carriers in ALI treatment.

**Figure 1:**
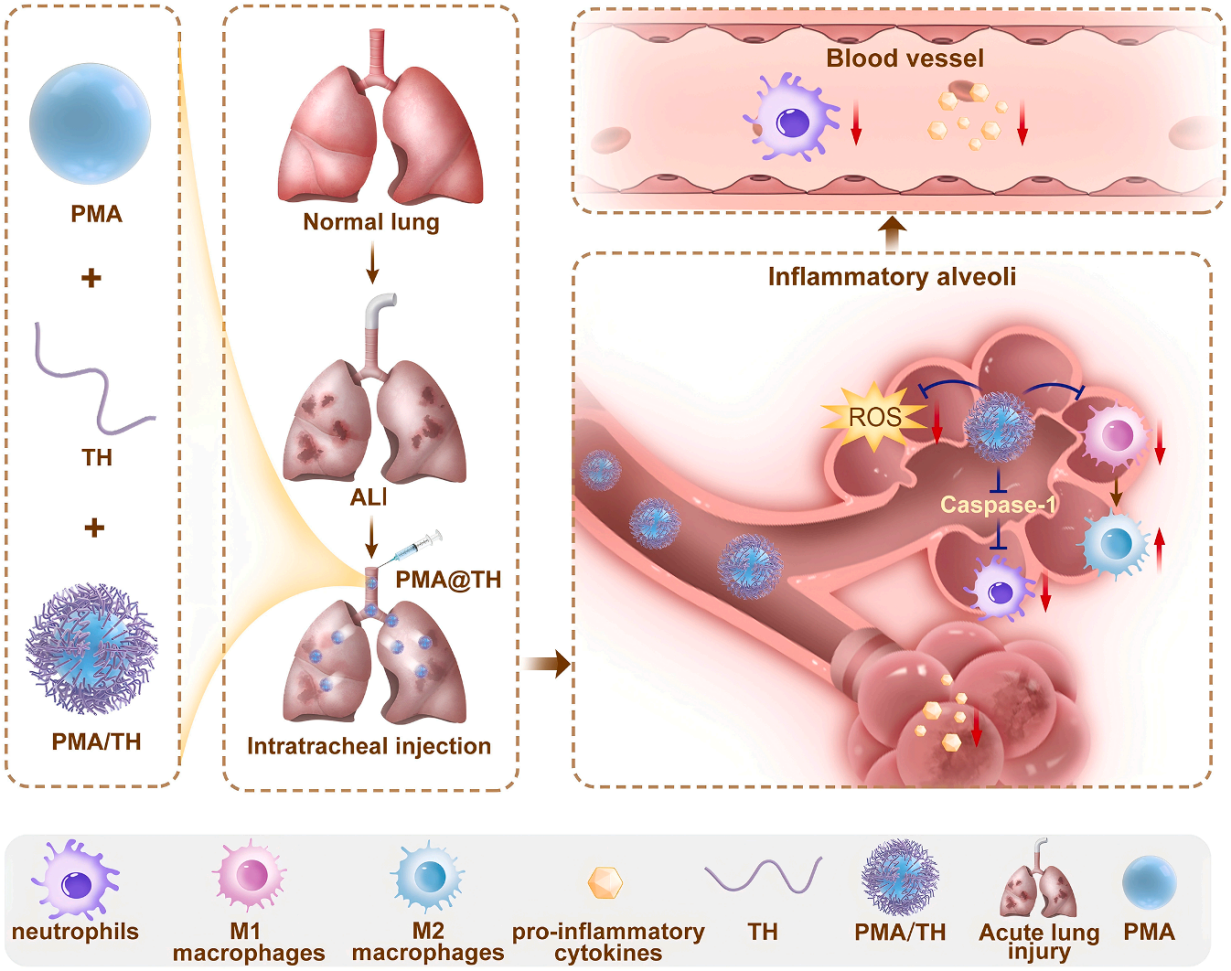
Schematic indicating the construction of PMA@TH nanomotor and its mechanism in ALI treatment

Presently, we developed an NO-driven nanomotor for ROS-targeted drug delivery, which was further combined with TH to achieve precise anti-inflammatory therapy for ALI (denoted as PMA@TH, seen in Figure 1). Upon intratracheal administration, the released NO propels the nanomotor toward injured sites with high ROS concentrations, enabling selective accumulation at inflammatory loci. Additionally, the superior hydrophilicity of PMA@TH enhances the therapeutic efficacy of TH, which acts by suppressing the caspase-1-IL-1β signaling pathway to inhibit neutrophil activation and infiltration, and further promoting the M2 activation of macrophages. Furthermore, PMA@TH mitigates systemic inflammation through alveolo-circulatory communication, as evidenced by the significantly reduced levels of pro-inflammatory cells and cytokines in circulation. Transcriptome sequencing further confirmed the anti-inflammatory and antioxidant effects of PMA@TH in ALI, highlighting its strong potential for clinical translation.

## Results

### Characterization and motion analysis of PMA@TH nanomotors

First, the methacrylamidated arginine monomer (MA) (Figure S1) was successfully synthesized. The characteristic peaks of the two hydrogens on the C=C bond observed in the MA nuclear magnetic resonance (NMR) spectrum (Figure S2) confirmed the successful double-bond modification. Subsequently, PMA nanomotors were synthesized via free radical polymerization using MA and crosslinker N, N′-bis(acryloyl)cystamine (BAC) as reactants with the addition of an initiator (Figure S3).

**Figure 2:**
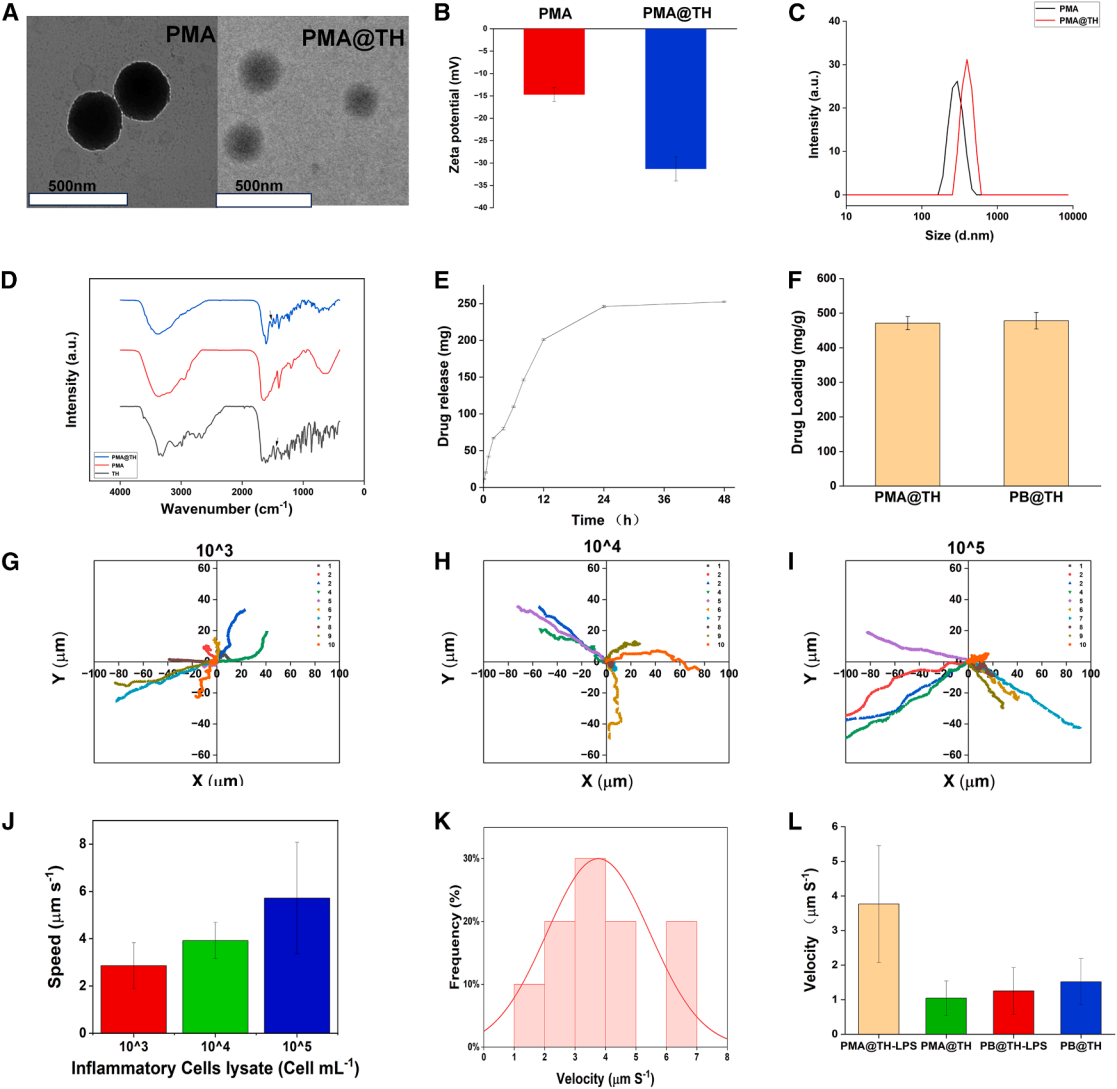
Characterization and motion analysis of PMA@TH nanomotors (A–C) (A) Transmission electron microscopy images (scale bars, 500 nm), (B) zeta potential, and (C) dynamic light scattering results. (D) FTIR spectra of PMA@TH, PMA, and TH (*n* = 3 independent sample preparations per group). (E) Cumulative TH release from PMA@TH in PBS over 48 h (*n* = 3 independent sample preparations). (F) Drug loading capacities of PMA@TH and PB@TH (*n* = 3 independent sample preparations). (G–J) The motion trajectories and corresponding average rate graphs of nanomotors under different cell density lysis buffers. (K) Velocity distribution of PMA@TH under LPS stimulation. (L) Average velocities of PMA@TH and PB@TH in stimulated and non-stimulated RAW 264.7 macrophage environments (*n* = 10 nanoparticles tracked per group).

To compare the enhanced therapeutic effects contributed by motility, a structurally similar but non-motile control sample (PB) was prepared by self-crosslinking of BAC. PB exhibited similar particle size and surface charge to PMA (Figure S4). The drug (TH) was then loaded onto PMA nanomotors via electrostatic adsorption. As shown in Figures 2A–2C, the PMA nanomotors had an average diameter of 220 nm and a zeta potential of −14.7 ± 1.6 mV. After drug loading, the diameter increased to 330 nm and the zeta potential shifted to −31.3 ± 2.7 mV. Fourier transform infrared (FTIR) spectra (Figure 2D) revealed that the peaks at 1,400 and 1,650 cm^−1^ in PMA corresponded to C=O stretching vibrations, C=C stretching vibrations, and N–H bending vibrations. A distinct peak at 1,503 cm^−1^ observed in PMA@TH, absent in PMA, was attributed to the aromatic rings in TH, preliminarily confirming successful drug loading.

To quantify TH loading, a standard curve of TH at 360 nm was established (Figure S5). As shown in Figure 2E, the drug loading capacities of PMA and PB were 471 mg/g and 478 mg/g, respectively. Drug release profiles in PBS (Figure 2F) demonstrated sustained release of 252 mg of TH from PMA@TH over 48 h. The mechanism underlying the drug release was also investigated, and we adopted three classical models to simulate its release curve. As indicated in Figure S6, the release simulation of PMA@TH is more in line with the Peppas model. The corresponding parameters were organized in Table S1. Moreover, the drug release profile results with PMA as the control group were also shown in Figure S6.

To evaluate the motion capability of the nanomotors, the trajectories of PMA@TH and PB@TH were tracked under stimulated and non-stimulated macrophage environments (Figures S7–S9). The highly expressed iNOS and ROS in the inflammatory microenvironment are important principles driving the movement of nanomotors. Therefore, we conducted a detailed study on the movement of nanomotors under different concentrations of iNOS and ROS. Cell lysates under inflammatory stimulation conditions of different densities were selected as the sources of iNOS and ROS. The greater the cell density, the higher the concentrations of iNOS and ROS. The results are shown in Figures 2G–2I—when the cell density increases from 10^3^ to 10^5^, the movement speeds of the nanomotor are 2.8, 3.9, and 5.7 μm s^−1^, respectively (Figure 2J). The velocity distributions (Figures 2K–2L; Figure S10; Videos S1, S2, S3, and S4) revealed that PMA@TH exhibited an average velocity of 3.8 μm s^−1^ under lipopolysaccharide (LPS)-stimulated RAW 264.7 macrophages, which was 2.9 times higher than that of PB@TH (1.3 μm s^−1^). In contrast, both PMA@TH and PB@TH showed negligible differences in velocity under non-stimulated conditions, displaying Brownian motion. These results indicate that PMA@TH nanomotors exhibit enhanced motility specifically in inflammatory environments.

Video S1. Velocities of PB@TH in non-stimulated RAW 264.7 macrophage environments Description of Figure S10.

Video S2. Velocities of PB@TH in stimulated RAW 264.7 macrophage environments Description of Figure S10.

Video S3. Velocities of PMA@TH in non-stimulated RAW 264.7 macrophage environments Description of Figure S10.

Video S4. Velocities of PMA@TH in stimulated RAW 264.7 macrophage environments Description of Figure 2.

### PMA@TH relieves the inflammatory lung injury of ALI mice

**Figure 3:**
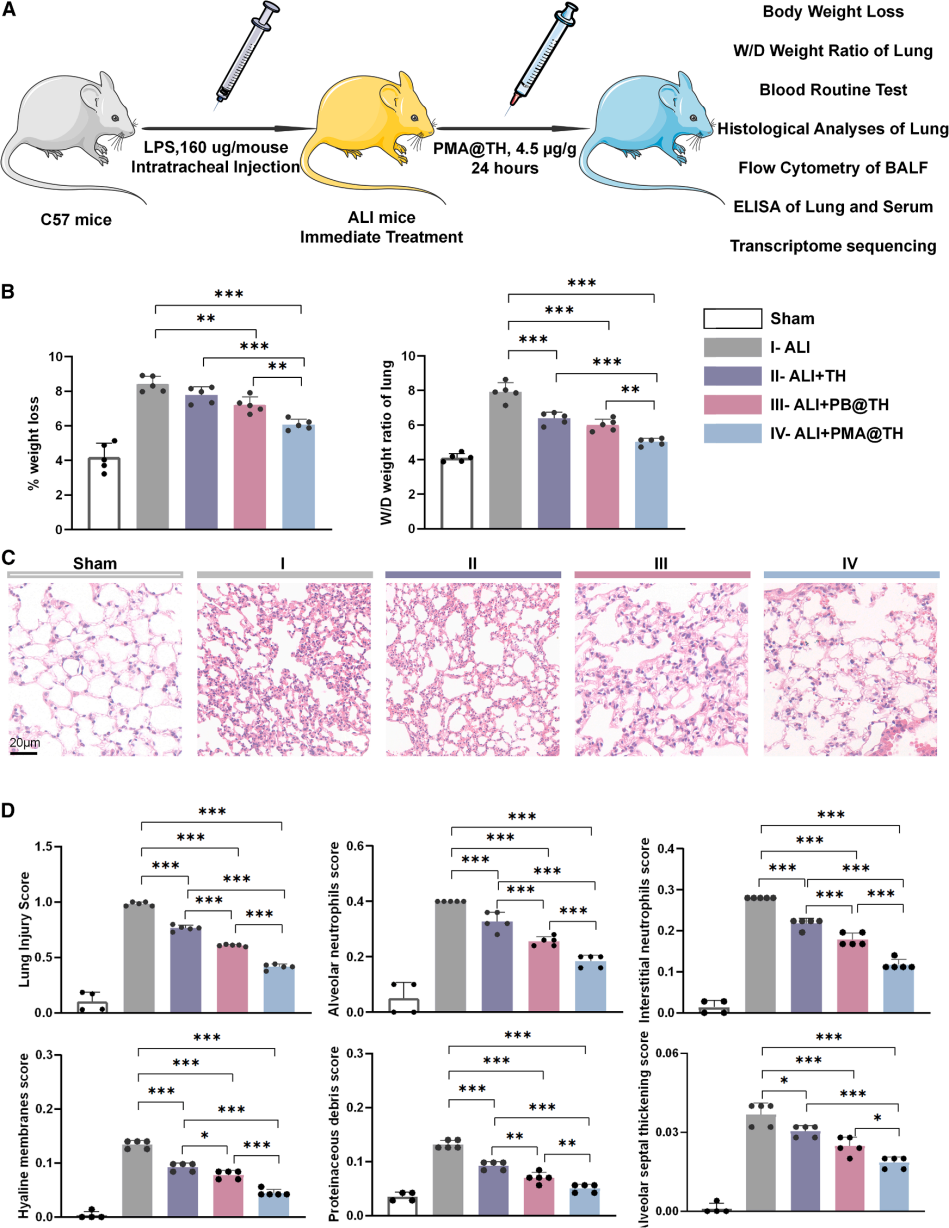
PMA@TH relieves inflammatory lung injury in ALI mice (A) Experimental design and treatment schedule. (B) Percentage of body weight loss (left) and lung wet-to-dry (W/D) weight ratio (right) in different treatment groups (*n* = 5 biologically independent mice per group). (C) Representative H&E-stained lung sections (scale bar, 20 μm). (D) Lung injury scores quantified from histology (*n* = 5 biologically independent mice per group). Statistical analysis: one-way ANOVA followed by Tukey’s multiple comparisons test. Data are shown as mean ± SD. ∗*p* < 0.05, ∗∗*p* < 0.01, ∗∗∗*p* < 0.001. Groups: I, ALI; II, ALI + TH; III, ALI + PB@TH; IV, ALI + PMA@TH.

To assess the therapeutic efficacy of PMA@TH *in vivo*, an ALI model was established via intratracheal LPS administration. Control mice received an equal volume of PBS instead. After LPS administration, the ALI mice were immediately treated with TH, PB@TH, or PMA@TH to evaluate their therapeutic effects. After 24 h, the mice were weighted and then euthanized, and the left lung lobes were collected for wet-to-dry (W/D) weight ratio and histological analysis of lung injuries (Figure 3A).

As illustrated in Figure 3B, the ALI mice exhibited a significant reduction in body weight, suggesting a systemic illness. Further, the W/D weight ratio of lung, a measurement of pulmonary edema, was markedly elevated in the ALI mice. Treatments with TH, PB@TH, or PMA@TH mitigated the injury-induced body weight loss and substantially reduced the lung W/D ratio. Notably, PMA@TH showed the most significant treatment efficiency. Consistently, according to H&E staining, LPS administration induced severe lung injury, evidenced by a significant increase in all the five independent features, including alveolar neutrophils, interstitial neutrophils, hyaline membranes, proteinaceous debris filling airspaces, and alveolar septal thickening (Figures 3C and 3D). Furthermore, treatment with TH, PB@TH, or PMA@TH resulted in marked reductions in all the above-mentioned features (all *p* < 0.05), indicating the significant recovery from inflammatory lung injury. Notably, PMA@TH showed superior therapeutic outcomes compared to PB@TH and TH alone (all *p* < 0.05, Figure 3D). Furthermore, to further support the lung-protective and anti-inflammatory effects of TH@PMA, we employed the cecal ligation and puncture (CLP) model to induce indirect lung injury. As shown in Figure S11, CLP mice developed significant pulmonary inflammatory injury. Treatment with TH@PMA conspicuously attenuated these alterations.

### PMA@TH inhibits systemic inflammation in ALI mice

Systemic inflammation plays a critical role in ALI progression, primarily characterized by excessive inflammatory cell activation and pro-inflammatory cytokine release. To evaluate the impact of PMA@TH on these processes, we performed routine blood test to analyze leukocyte activation and multiplex ELISA to measure cytokine levels.

**Figure 4:**
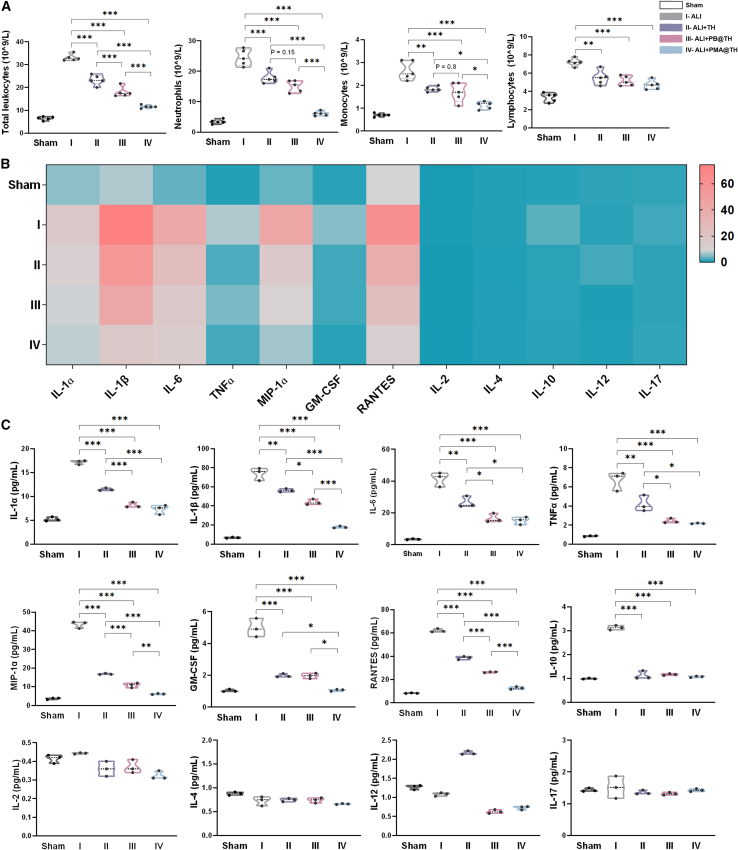
PMA@TH inhibits the systemic inflammation in ALI mice (A) Routine blood test of ALI mice (*n* = 5 biologically independent mice per group). (B) Heatmap of multiple inflammatory cytokines in the serum of ALI mice (*n* = 3 biologically independent mice per group). (C) Quantification of cytokines revealed by the multiplex ELISA (*n* = 3 biologically independent mice per group). Statistical analysis: one-way ANOVA followed by Tukey’s multiple comparisons test. Data are shown as mean ± SD. ∗*p* < 0.05, ∗∗*p* < 0.01, ∗∗∗*p* < 0.001.

As depicted in Figure 4A, ALI mice exhibited a substantial rise in blood leukocytes, which was markedly reduced by the treatment with TH, PB@TH, or PMA@TH (all *p* < 0.05). Notably, the PMA@TH group demonstrated significantly lower leukocyte counts compared to the TH and PB@TH groups (both *p* < 0.05, Figure 4A), which was mainly attributed to the decrease of neutrophils. Similarly, the level of monocytes and lymphocytes showed a comparable trend upon nanocomplex treatment, but differences among the three nanocomplex-treated groups were not always statistically significant (*p* > 0.05, details seen in Figure 4A).

Multiplex ELISA analysis (Figure 4B) revealed that following LPS treatment, the production of pro-inflammatory cytokines significantly increased in the serum of ALI mice, including IL-1ɑ, IL-1β, IL-6, TNF-ɑ, MIP-1ɑ, and RANTES. Upon nanocomplex administration, the expression of pro-inflammatory cytokines was markedly suppressed in ALI mice (all *p* < 0.05). Notably, PMA@TH exhibited even greater suppression than either TH or PB@TH (*p* < 0.05; Figure 4C). These findings collectively demonstrate that the combined PMA@TH therapy exerts superior anti-inflammatory effects over individual TH treatment, effectively attenuating both neutrophils activation and circulating cytokine production.

Regarding the CLP-induced ALI mice, TH@PMA also significantly mitigated systemic inflammation, as indicated by the reduced peripheral leukocyte/neutrophil counts (Figure S12) and the lower pro-inflammatory cytokine levels (Figure S13). In contrast to the LPS-induced ALI mice, TH and PB@TH showed minimal effect on systemic inflammation in the CLP model. This discrepancy is logical and can be attributed to the distinct pathophysiology of the CLP model and the route of drug administration.

### PMA@TH suppresses local inflammation in ALI mice

**Figure 5:**
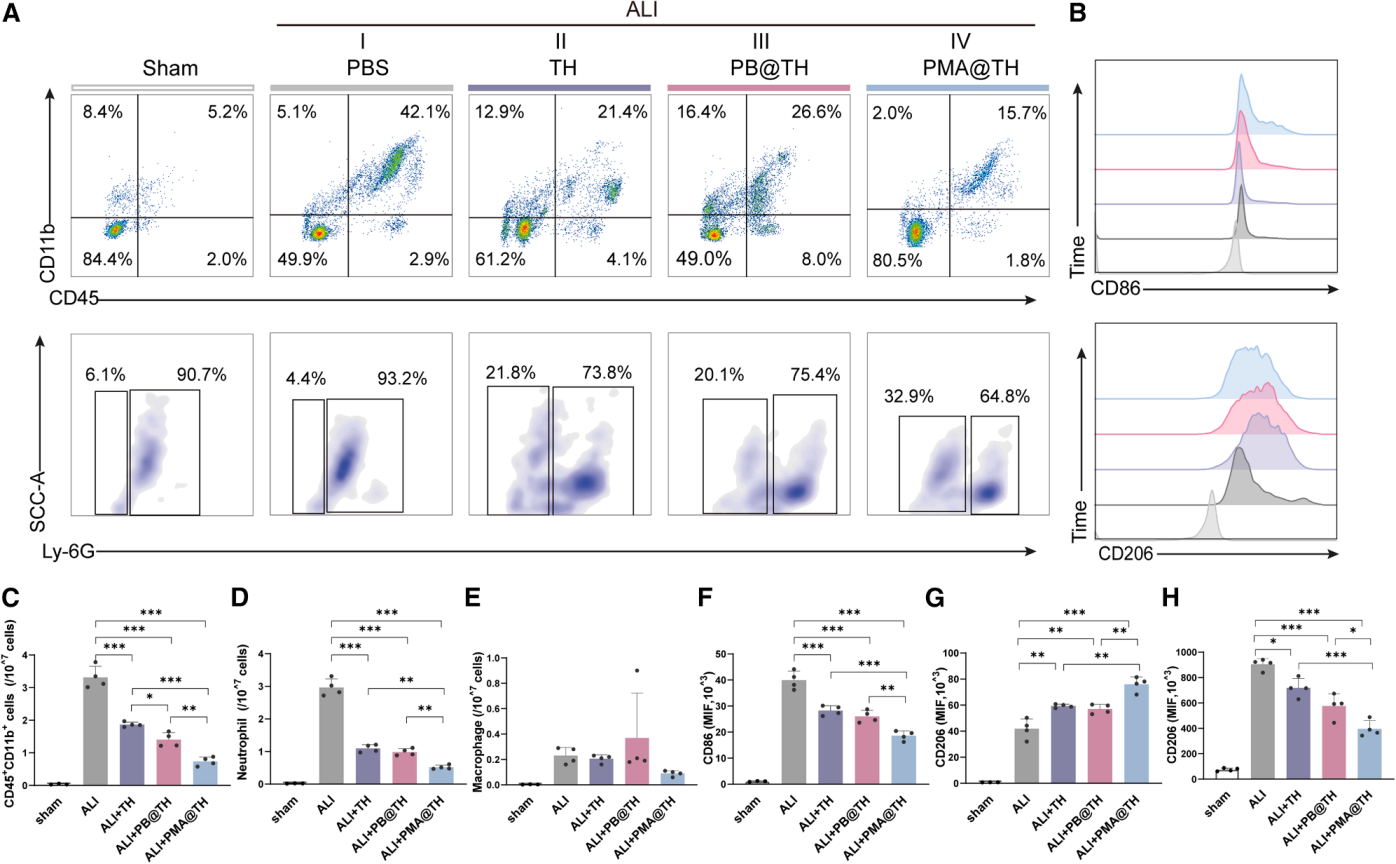
PMA@TH suppresses the local inflammation in the BALF of ALI mice (A) Flow cytometric images of CD45^+^ CD11b^+^ inflammatory cells, neutrophils, and macrophages in the BALF of ALI mice. (B) Flow cytometric images of CD86 and CD206 MFIs of macrophages in the BALF. (C–E) Quantification of inflammatory cells in the BALF (*n* = 4 biologically independent mice per group). (F and G) The quantification of CD86 and CD206 MFIs in the BALF (*n* = 4 biologically independent mice per group). (H) The concentration of total protein in BALF (*n* = 4 biologically independent mice per group). Statistical analysis: one-way ANOVA followed by Tukey’s multiple comparisons test. Data are shown as mean ± SD. ∗*p* < 0.05, ∗∗*p* < 0.01, ∗∗∗*p* < 0.001.

To further assess pulmonary inflammation, we analyzed the immune cell infiltration and the total protein level in the bronchoalveolar lavage fluid (BALF). The results demonstrated that the nanocomplexes markedly decreased CD45^+^CD11b^+^ inflammatory cell levels in BALF (see Figure S14 for gating strategy), primarily due to neutrophil suppression (Figures 5A–5D). While macrophage counts remained unaffected (Figure 5E), the PMA@TH notably enhanced M2 polarization, as evidenced by the elevated CD206 mean fluorescence intensity (MFI). Additionally, PMA@TH also significantly suppressed M1 macrophage activation, as indicated by the decreased CD86 MFI (Figures 5B, 5F, and 5G). Consistently, all the aforementioned nanocomplexes also significantly reduced the total protein concentration in the BALF of ALI mice, with PMA@TH demonstrating superior therapeutic efficacy compared to TH and PB@TH (Figure 5H).

**Figure 6:**
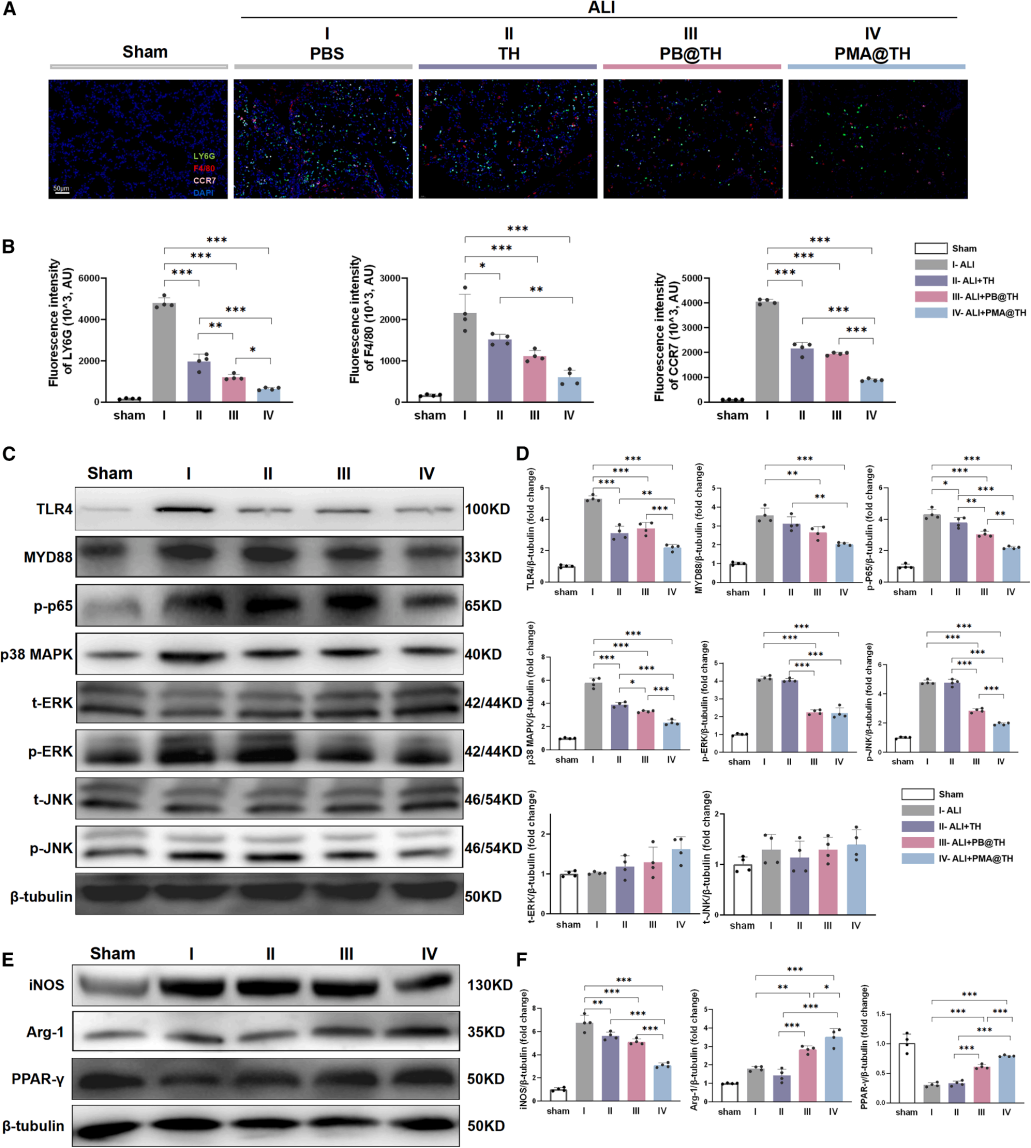
PMA@TH suppresses local inflammation in lung tissue of ALI mice (A and B) Representative immunofluorescence images and quantification of LY6G, F4/80, and CCR7 in lung tissue (*n* = 4 biologically independent mice per group). (C–F) Representative western blot images and quantification of inflammatory signaling in lung tissue (*n* = 4 biologically independent mice per group). Statistical analysis: one-way ANOVA followed by Tukey’s multiple comparisons test. Data are shown as mean ± SD. ∗*p* < 0.05, ∗∗*p* < 0.01, ∗∗∗*p* < 0.001.

Regarding the lung tissue, the expression of cytokines, the infiltration of immune cells, and the activation of inflammatory signaling pathways were also evaluated. The immunofluorescence analysis indicated that the nanocomplexes showed a similar inhibition effect, as the MFI of immune cell markers, including LY6G, F4/80, and CCR7, in ALI mice was significantly decreased upon TH, PB@TH, or PMA@TH treatments (Figures 6A and S15). Consistently, PMA@TH exhibited stronger inhibition effects as compared to TH and PB@TH (Figure 6B). Moreover, the inflammatory cytokine profiles followed comparable trends (Figure S16). All treatment groups demonstrated significant suppression of cytokine expression, with PMA@TH showing superior therapy efficacy. To further elucidate the effect of nanocomplexes on classical pro-inflammatory pathways, we examined the activation of the TLR4/MYD88/p-NF-κB p65 and MAPK/ERK/JNK pathways in lung tissues by western blot. As shown in Figures 6C and 6D, these pathways were significantly activated in the lungs of ALI mice. All the nanocomplexes markedly attenuated this activation, as evidenced by the reversal of elevated TLR4, MYD88, p-NF-κB p65, and MAPK expressions, along with the reduced phosphorylation of ERK and JNK. Notably, the PMA@TH nanocomplex demonstrated the most potent inhibitory effect.

To further elucidate the underlying mechanism of the macrophage phenotype shift, we investigated the M2 polarization markers and their upstream regulators via western blot (Figures 6E and 6F). Upon ALI induction, the expression of iNOS was markedly upregulated, accompanied by a significant suppression of the transcription factor PPAR-γ, while the expression of the M2 marker Arg-1 also exhibited a slight elevation in the ALI group compared to the sham mice. Following treatment with nanocomplex, the expression of iNOS was substantially reduced, whereas the levels of PPAR-γ and Arg-1 were significantly upregulated. Of note, PMA@TH exhibited the most potent treatment effects as compared to TH and PB@TH (Figure 6F). Considering that excessive iNOS-derived oxidative and nitrosative stress heavily impairs tissue repair, we postulate that the effective inhibition of iNOS by the nanomotor creates a favorable microenvironment that facilitates M2 polarization. Collectively, these results demonstrate that PMA@TH facilitates macrophage polarization toward an anti-inflammatory M2 phenotype, possibly by inhibiting iNOS and reactivating PPAR-γ signaling, which may also contribute to the observed suppression of the TLR4 pathway. Collectively, while all three nanocomplex formulations, namely, TH, PB@TH, and PMA@TH, effectively attenuated local inflammation in ALI mice, the combined PMA@TH treatment consistently produced the most robust anti-inflammatory response.

### Mechanistic insights into the therapeutic effects of PMA@TH in ALI

**Figure 7:**
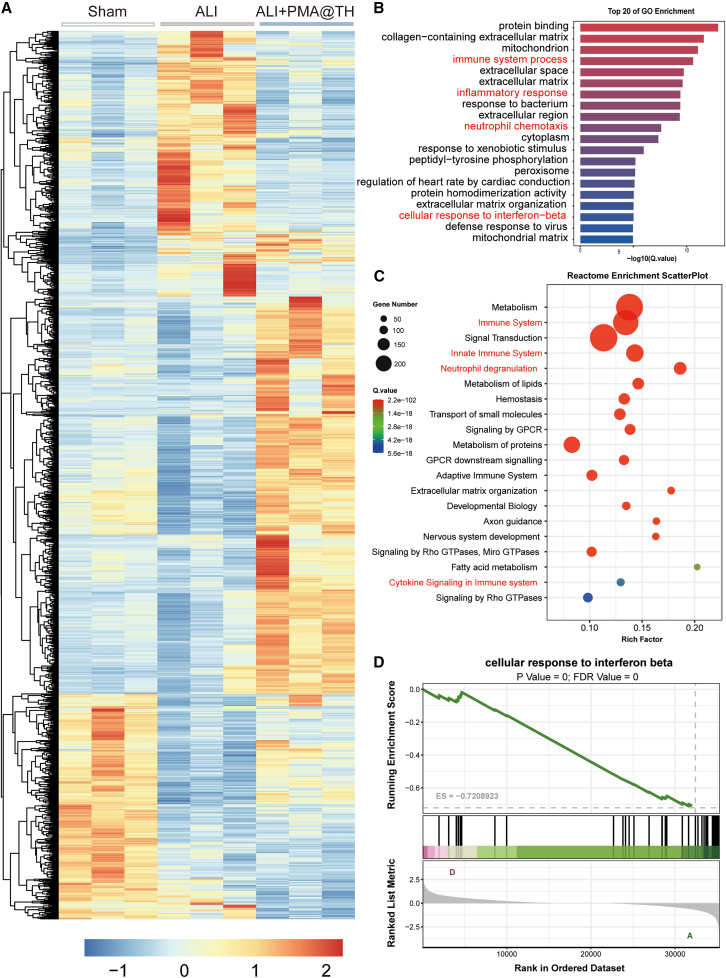
Mechanistic insights into the therapeutic effects of PMA@TH in ALI (A) Alterations of lung tissue transcription in the PMA@TH-treated ALI mice (*n* = 3 biologically independent mice per group). (B and C) GO and Kyoto Encyclopedia of Genes and Genomes analyses show the most downregulated pathways in ALI mice under PMA@TH treatment. (D) GSEA highlights the significant inhibition of IFN-β signaling in the PMA@TH-treated ALI mice.

To elucidate the protective mechanisms of PMA@TH in ALI, RNA sequencing was performed on inflammatory lung tissue. Treatment with PMA@TH induced significant transcriptional changes in ALI mice (Figure 7A). Comparative analysis revealed 1,872 differentially expressed genes (DEGs) in the ALI mice versus the PMA@TH-treated ALI mice, with 1,106 upregulated and 762 downregulated genes.

To further explore the anti-inflammatory mechanisms of PMA@TH, Gene Ontology (GO), Reactome analysis, and gene set enrichment analysis (GSEA) were conducted (Figures 7B–7D). As anticipated, DEGs between the ALI and treatment groups were predominantly enriched in inflammation-related pathways, including immune system process, inflammatory responses, neutrophil chemotaxis, cytokine signaling, and chemokine-mediated signaling (Figures 7B and 7C). Additionally, response to INF-β signaling was also highlighted by the Reactome analysis (Figure 7D).

To conclude, transcriptomic analysis demonstrated that PMA@TH mitigates ALI by suppressing neutrophilic activation, INF-β-induced immune response and chemokine-driven inflammation, thereby protecting against systemic and localized inflammatory damage.

## Discussion

ALI is characterized by severe alveolar inflammation, oxidative stress, and a massive influx of neutrophils. Currently, specific pharmacological interventions for ALI remain limited. In this study, we developed an NO-driven nanomotor (PMA@TH) to actively target the injured lung microenvironment. Our results indicate that this system effectively delivers TH to inflammatory loci, reduces oxidative stress, and mitigates both local and systemic inflammation.

The first major advantage of our design is its active targeting and motion capability within the complex inflammatory microenvironment. Traditional passive drug delivery systems often struggle to penetrate dense alveolar exudates. By utilizing an ROS-responsive mechanism, PMA@TH turns a core pathological feature of ALI—excessive oxidative stress—into a driving force. Upon encountering the high-ROS environment of the injured lung, the nanomotors generate NO, propelling themselves deeper into the tissue. This process not only increases TH accumulation at the specific sites of injury but also directly consumes harmful ROS. Consequently, it interrupts the cycle of oxidative damage that normally degrades the alveolar-capillary barrier.

Beyond targeted drug delivery, PMA@TH actively modulates local immune responses. During the early stages of ALI, overactivation of the TLR4/MYD88/NF-κB signaling axis triggers a cytokine storm. Our data show that PMA@TH not only effectively inhibited these pro-inflammatory pathways but also promoted the transition of macrophages from an inflammatory M1 activation to an anti-inflammatory M2 activation. As supported by our protein analyses, PMA@TH treatment significantly upregulated the expression of the upstream transcription factor PPAR-γ and the downstream M2 marker Arg-1, while profoundly suppressing the M1 marker iNOS. The observed reduction in TLR4 expression and the subsequent M2 macrophage polarization may be partially attributed to the restoration of PPAR-γ. Previous studies have shown that excessive oxidative stress during ALI impairs PPAR-γ function, leading to overactive TLR4 signaling. By efficiently scavenging ROS and releasing NO, the PMA@TH nanomotor relieves the local oxidative burden and restores PPAR-γ expression in the injured lung. Once restored, PPAR-γ may exert a dual immunomodulatory effect. It has been reported to repress the TLR4/NF-κB axis to dampen the pro-inflammatory cascade,^29^ while potentially promoting the transcription of M2-associated repair genes like Arg-1.^30^ Furthermore, the significant downregulation of iNOS by the nanomotor reduces inflammatory metabolic stress, providing a more favorable microenvironment for this phenotype shift. Meanwhile, our GSEA analysis identified the suppression of IFN-β signaling after PMA@TH treatment. Given that type I interferon signaling promotes neutrophil recruitment and pro-inflammatory responses during ALI, this suppression partially explains the reduced neutrophil chemotaxis and attenuated cytokine production observed in our study. Furthermore, as IFN-β signaling engages in crosstalk with the TLR4/NF-κB axis, its suppression may synergize with the inhibition of classical inflammatory pathways, further contributing to the favorable microenvironment for M2 macrophage polarization. Taken together, PMA@TH effectively couples oxidative stress relief with PPAR-γ-associated macrophage activation.

From a translational perspective, PMA@TH offers a practical strategy to overcome the dose-limiting side effects of conventional ALI therapies. High systemic doses of anti-inflammatory drugs often lead to unwanted immunosuppression or secondary organ toxicity. Because PMA@TH releases its payload primarily in response to local ROS, systemic exposure to TH is kept to a minimum. Furthermore, routine blood tests and histological evaluations confirmed that the formulation did not induce obvious toxicity in the short term, highlighting the safety profile of this NO-driven nanotherapeutic approach.

### Limitations of the study

While our study demonstrates the therapeutic potential of PMA@TH nanomotors, several limitations exist. First, we conducted the *in vivo* experiments exclusively on male mice to exclude the influence of female hormonal fluctuations on inflammation. This lack of sex as a biological variable limits the generalizability of our findings, making it necessary to include both sexes in future studies.^31^^,^^32^ Second, the sample sizes are relatively small. Although adequate for identifying statistical significance in this initial stage, larger animal cohorts are required to fully confirm these results. Third, our therapeutic assessment was limited to a 24-h acute window. The long-term systemic toxicity, biodistribution, and clearance pathways of the nanomotors have yet to be determined. Last, since the LPS and CLP mouse models cannot perfectly replicate the complexity of clinical acute respiratory distress syndrome, further validation in larger animal models is needed before translation.

In conclusion, the PMA@TH nanomotor provides an active and targeted strategy for treating ALI. By combining ROS scavenging, localized drug release, and immune microenvironment regulation, it effectively limits alveolar damage and systemic inflammation, offering a promising platform for future nanomedicine development.

## Resource availability

### Lead contact

Requests for further information and resources should be directed to and will be fulfilled by the lead contact, Fan Su (13057565195@163.com).

### Materials availability

All unique/stable reagents generated in this study are available from the lead contact with a completed materials transfer agreement.

### Data and code availability

• All data reported in this paper will be shared by the lead contact upon request.
• The raw RNA-seq data reported in this paper have been deposited in the Genome Sequence Archive (GSA, https://ngdc.cncb.ac.cn/gsa) under accession number CRA045199 and are publicly available as of the day of publication.
• This paper does not report original code.
• Any additional information required to reanalyze the data reported in this paper is available from the lead contact upon request.

## Acknowledgments

We greatly acknowledge the financial support from the Fundamental Research Funds for the Central Universities, grant nos. YG2023QNB25 (to F.S.), YG2024QNA52 (to Q.Z.), and YG2023QNA40 (to C.Z.); Shanghai Municipal Health Commission Talent Program, grant no. 2002YQ022 (to F.S.); and 10.13039/501100001809National Natural Science Foundation of China, grant no. 82470360 (to J.H.).

## Author contributions

Y. Sun, F.S., and J.H. designed the experiments; S.L., Z.Z., Q.Z., C.Z., and Y. Shen performed the experiments; J.H., Y. Sun, and F.S. carried out the data analysis and wrote the manuscript. Y. Sun and F.S. supervised the research. All authors proofread and approved the final version of this manuscript for submission. S.L., Z.Z., and Q.Z. contributed equally to this work.

## Declaration of interests

The authors declare no competing financial interests.

## Declaration of generative AI and AI-assisted technologies in the writing process

During the preparation of this work the author used *Deepseek* to improve the readability and language of the manuscript. After using this tool, the authors reviewed and edited the content as needed and took full responsibility for the content of the published article.

## STAR★Methods

### Key resources table

**Table:** 

REAGENT or RESOURCE	SOURCE	IDENTIFIER
**Antibodies**		

IL-1β	Abmart	RRID: AB_2936800
IL-6	Abmart	RRID: AB_2936896
TNFα	Abmart	RRID: AB_2920645
F4/80	Abcam	RRID: AB_2936298
Ly-6G (Immunofluorescence)	Proteintech	RRID: AB_2918382
CCR7	Proteintech	RRID: AB_2880289
TLR4	Proteintech	RRID: AB_10638446
MYD88	Servicebio	RRID: AB_3186235
Phospho-NF-kB p65	Servicebio	Cat# GB153882; RRID: AB_3753373
p38 MAPK	Cell Signaling Technology	RRID: AB_10999090
ERK1+ERK2	Servicebio	RRID: AB_3717515
Phospho-ERK1 + ERK2	Servicebio	RRID: AB_3714747
JNK	Servicebio	RRID: AB_3714563
Phospho-JNK	Servicebio	RRID: AB_3717516
β-Tubulin	Proteintech	RRID: AB_2210695
Ly-6G (flow cytometry)	BioLegend	RRID: AB_10897944
CD206	BioLegend	RRID: AB_10896057
CD86	BioLegend	RRID: AB_439783
CD45	BioLegend	RRID: AB_893339
CD11b	BioLegend	RRID: AB_312788
iNOS	Proteintech	RRID:AB_2782960
PPAR-γ	Proteintech	RRID:AB_2882260
Arg-1	Proteintech	RRID:AB_2289842

**Chemicals, peptides, and recombinant proteins**		

L-arginine	Yuanye Biotechnology	Cat# B21920
Triethylamine	Aladdin Chemistry	Cat# T103287
methacrylic anhydride	Aladdin Chemistry	Cat# M1500602
ammonium persulfate	Shanghai Macklin Biochemical Technology	Cat# A801034
TEMED	Shanghai Aladdin Biochemical Technology	Cat# T105497
Tetracycline hydrochloride	MedChemExpress	Cat# HY-B0474
LPS	Shanghai Yeasen Biotechnology	Cat# 60747ES08
LIVE/DEAD™	Thermo	Cat# L34955
TRIzol Reagent	Thermo	Cat# 15596018CN
BCA	Macklin	Cat# 60984-57-8
EDC	Aladdin	Cat# 25952-53-8
NHS	Shanghai yuanye Bio-Technology	Cat# 6066-82-6
Cy5-NHS ester	Duofluor	Cat# 1032678-42-4
sodium pentobarbital	Merck	Cat# P3761

**Critical commercial assays**		

Multiplex ELISA Kit	Boster Bro	Cat# MEK1003
BCA Protein Assay Kit	Beyotime	Cat# P0012
RIPA Lysis Buffer	Beyotime	Cat# P0013B

**Deposited data**		

RNA-seq data	GSA, https://ngdc.cncb.ac.cn/gsa	CRA045199

**Experimental models: Cell lines**		

RAW 264.7 macrophages	Procell	RRID: CVCL_0493

**Experimental models: Organisms/strains**		

C57BL/6J (Male, 8–12 weeks old)	Shanghai Model Organisms Center	Cat# SM-001

**Software and algorithms**		

Statistical Product and Service Solutions	IBM	RRID: SCR_002865
FlowJo software	Becton, Dickinson & Company	RRID: SCR_008520
DESeq2	SciCrunch Registry	RRID: SCR_015687
ImageJ	National Institutes of Health	RRID: SCR_003070
ChemDraw	PerkinElmer	RRID:SCR_016768

### Experimental model and study participant details

#### Animal models

Male C57BL/6J mice (8–12 weeks old, 20–25 g body weight) were purchased from Shanghai Model Organisms Center (Shanghai, China). All animal experiments were performed in accordance with relevant institutional and national guidelines and regulations, and were approved by the Animal Care and Use Committee of Shanghai Chest Hospital (KS(Y)26138).The mice were group-housed (six mice per cage) under specific pathogen-free (SPF) conditions at a standard temperature of 22 ± 2°C, a humidity of 50 ± 10%, and a 12-h light/dark cycle, with free access to standard food and water. Animals were randomly allocated to experimental groups using a random number generator and the investigators were blinded during outcome assessment. All mice used in this study were drug- and test-naive and had not undergone any previous procedures prior to the experiments.

Limitation regarding sex: In this study, only male mice were used to exclude the fluctuations of female hormones on the inflammatory response in ALI. The absence of female subjects is a limitation to the generalizability of our findings, and future studies including both sexes will be necessary to determine if there are sex-dependent differences in the therapeutic efficacy of PMA@TH nanomotors.

#### Cell lines

The murine macrophage cell line RAW 264.7 (RRID: CVCL_0493) was obtained from Procell. Cells were cultured in Dulbecco’s Modified Eagle Medium (DMEM) supplemented with 10% fetal bovine serum (FBS). RAW 264.7 is a mouse male-derived cell line. Cells were authenticated by STR profiling and routinely tested for mycoplasma contamination by luminescence kit before experiments.

### Method details

#### Synthesis of L-Arginine derivative

First, L-arginine (L-Arg, 2.0 g, 11.5 mmol, Yuanye Biotechnology Co., Ltd.) was dissolved in a mixed solution of distilled water (20.0 mL) and 1,4-dioxane (8.5 mL, Sinopharm Chemical Reagent Co., Ltd.). Triethylamine (4.5 mL, 32.3 mmol, Aladdin Chemistry Co., Ltd.) was then added dropwise to the mixture. After cooling the reaction system in an ice-water bath, methacrylic anhydride (3.0 mL, 18.9 mmol, Aladdin Chemistry Co., Ltd.) was slowly added dropwise under stirring over 10 min. The ice-water bath was removed, and the reaction was stirred overnight at room temperature. The product was reprecipitated twice by adding it to 400.0 mL of acetone, followed by vacuum drying at 60°C to obtain the final product, named methacrylated arginine (M-Arg), abbreviated as MA.

#### Synthesis of PMA nanomotors

First, BAC (2.00 mg, 0.0077 mmol, Shanghai Macklin Biochemical Technology Co., Ltd.) and MA (9.25 mg, 0.0385 mmol) were dissolved in phosphate-buffered saline (PBS, 2.0 mL) and ultrasonically mixed. The solution was purged with nitrogen for 30 min. Subsequently, the initiators, ammonium persulfate (APS, 4% of the system, 0.45 mg, Shanghai Macklin Biochemical Technology Co., Ltd.) N, N, N′, N′-Tetramethylethylenediamine (TEMED, 8% of the system, 0.90 mg, Shanghai Aladdin Biochemical Technology Co., Ltd.) were added under continuous nitrogen flow. The reaction was stirred at room temperature for 2 h until the solution turned turbid. The product was centrifuged (8000 rpm, 10 min), washed twice with water, and lyophilized to obtain a white solid.

#### Synthesis of non-nanomotor PB

BAC (12.00 mg, 0.0462 mmol) was dissolved in PBS (2.0 mL) and ultrasonically mixed. The solution was purged with nitrogen for 30 min. Initiators APS (4% of the system, 0.48 mg) and TEMED (8% of the system, 0.96 mg) were added under continuous nitrogen flow. The reaction was stirred at room temperature for 2 h until turbidity appeared. The product was centrifuged (8000 rpm, 10 min), washed twice with water, and lyophilized to yield a white solid, which is denoted as PB non-nanomotor.

#### Preparation of PMA@TH nanomotor

PMA (20 mg) was dispersed in a TH-containing PBS solution (5 mL, 2.5 mg/mL) and stirred at room temperature for 12 h. After centrifugation, the white solid was collected and named as PMA@TH Nanomotor.

#### Preparation of TH standard curve

Tetracycline hydrochloride (TH, MedChemExpress Co., Ltd.) was dissolved in PBS to prepare solutions with concentrations of 50, 25, 10, 4, 2, and 1 mg/L. The absorbance at 360 nm was measured using a fully automated microplate reader (Tecan Trading Co., Ltd., Shanghai). A standard curve of TH concentration versus absorbance was plotted.

#### Determination of drug loading capacity

PMA (20 mg) was dispersed in a TH-containing PBS solution (5 mL, 2.5 mg/mL) and stirred at room temperature for 12 h. The supernatant was collected after centrifugation (13000 rpm, 10 min) and washed with 5 mL of deionized water. The absorbance of the supernatant was measured at 360 nm, and the TH content was calculated using the standard curve. The drug loading capacity (mg/g) was calculated as:

$$\text{Loading}\;\text{capacity}\;\left(\text{mg}/g\right)=\frac{{\text{TH}}_{\text{total}}-{\text{TH}}_{\text{supernatant}}}{m_{\text{PMA}}}$$

Where m_THtotal_ is the total mass of TH in solution (mg), m_THsupernatant_ is the residual TH mass in the supernatant (mg), and THm_PMA_ is the mass of PMA (g).

#### Drug release measurement

PMA@TH and PB@TH (2 mg each) were dispersed in PBS (2 mL) and incubated on a silent mixer at 37°C. At 0, 10, and 30 min, and 1, 2, 4, 8, 12, 24, and 48 h, samples were centrifuged at 10,000 rpm, and the supernatant was collected for absorbance measurement at 360 nm. An equal volume of fresh PBS was added back after each sampling to maintain sink conditions. Cumulative drug release was calculated using the TH standard curve. Three independent sample preparations were analyzed per group (*n* = 3).

#### Mechanism of drug release

To study its release mechanism, this paper adopts three classical models, namely the pseudo-zero-order dynamic model,^33^ the pseudo-first-order dynamic model^34^ and the Peppas (1987) model,^34^ to simulate its release curve. The formula of these drug release models is as follows.

(1) The zero-order kinetics equation:*Q=K*_*0*_*t+A*
(2) The first-order kinetics equation: *Ln (Q*_*0*_*-Q*_*t*_*)=LnQ*_*0*_*-K*_*1*_*t*
(3) Peppas equation: *Q*_*t*_*/Q*_*0*_ *= a ⋅ t*^*b*^

Where *Q*_*0*_ *r*epresents the total drug release amount, *Q*_*t*_ refers to the drug release amount at a specific time, and *k* and *A* as well as *a* and *b* are all constants in the model. For the Peppas model, to achieve better fitting, we simultaneously take ln on both sides of the equation to obtain lnQ_t_/Q_0_ = lna + b lnt and then plot it with *lnt* as the x-coordinate and *lnQt/Q0* as the y-coordinate, followed by fitting.

#### Synthesis of Cy5-Labeled samples

PMA@TH nanomotor and PB@TH non-nanomotor s were dispersed in PBS (2 mL, 1 mg/mL). EDC (2 mg, Shanghai Macklin Biochemical Technology Co., Ltd.) and NHS (2 mg, Shanghai Aladdin Biochemical Technology Co., Ltd.) were added and activated for 3 h Cy5-NHS ester (2 μL, Duofluor Biotechnology Co., Ltd.) was added, and the reaction was stirred overnight. The product was centrifuged (5800 × g, 8 min), washed three times with water, and lyophilized to obtain Cy5-labeled samples.

#### Material characterization

The nanoparticle morphology was investigated using an HT7800 transmission electron microscope (Hitachi, Japan). Zeta potential and particle size measurements were performed with a Nano-Z Zetasizer (Malvern Instruments, UK). ^1^H-NMR spectra of the samples were recorded on a Bruker Avance 400 spectrometer. Absorbance measurements at 360 nm were obtained using a fully automated microplate reader (Tecan (Shanghai) Experimental Equipment Co., Ltd.). FTIR spectra were acquired using a Cary 5000 FTIR spectrophotometer (Varian, USA). The motion tracking of nanomotors in cellular environments was captured through fluorescence microscopy (MF53-N, Guangzhou Micro-shot Technology Co., Ltd.). Chemical structural formulas were drawn using ChemDraw Ultra 7.0 software. n denotes the number of independent sample preparations analyzed for each measurement. For the measurements of TEM, DLS, zeta and FTIR, *n* = 3 independent preparations with three technical reads.

#### Analysis of motion behavior in cellular environments

RAW 264.7 macrophages (5×10^4^ cells mL^−1^) were seeded in a culture dish and incubated overnight. After stimulating macrophages with lipopolysaccharide (LPS, Shanghai Yeasen Biotechnology Co., Ltd.) for 12 h, Cy5-labeled PMA@TH and PB@TH (200 μg/mL) were added. As a control, the samples were also added to unstimulated macrophages. Motion trajectories were tracked using an inverted fluorescence microscope (×100 objective). Image sequences were analyzed with ImageJ (v2.0.0, RRID:SCR_003070). The mean speed of particles was calculated by averaging velocities over time intervals. Ten nanoparticles per group were tracked to determine mean speed and MSD values. MSD fitting was performed to classify particle motion types. To avoid ambiguity, n denotes the number of individual nanoparticles tracked per group. Specifically, *n* = 10 nanoparticles per group. Values were summarized at the nanoparticle level, and the three tracking runs were used as technical replicates.

#### ALI models induction and treatments

To comprehensively evaluate the therapeutic efficacy of the PMA@TH nanomotors, two distinct *in vivo* models of ALI were established: a direct LPS-induced model and an indirect polymicrobial sepsis-induced cecal ligation and puncture (CLP) model.

##### LPS-induced ALI model

The LPS-induced ALI model was induced according to the previous investigations.^35^ Mice were anesthetized via intraperitoneal injection of sodium pentobarbital (80 mg/kg). Subsequently, 160 μg of LPS dissolved in 70 μL of sterile PBS was administered via intratracheal instillation to induce severe pulmonary injury. Immediately following LPS challenge, mice were intratracheally treated with TH (1 μg/g), PB@TH (4.7 μg/g), or PMA@TH (4.5 μg/g), all dissolved in 80 μL of PBS, to evaluate their *in vivo* therapeutic efficiency. The sham group received an equivalent volume of PBS intratracheally. To evaluate systemic illness severity, the body weights of the mice were recorded at 24 h post-injection and compared with their baseline weights to calculate the percentage of body weight loss. Subsequently, the mice were euthanized, and peripheral blood, lung tissues, and bronchoalveolar lavage fluid (BALF) were harvested for subsequent analyses.

##### CLP-induced ALI model

To validate the systemic anti-inflammatory effects, the CLP model was performed. Briefly, a 1 cm longitudinal midline laparotomy was performed to carefully expose the cecum. The cecum was then isolated and tightly ligated at exactly the midpoint (1/2 of its length from the distal end) using a 4-0 silk suture to ensure a consistent and severe injury severity. Two punctures were performed through the ligated cecum using a 21-gauge needle, and a small amount of fecal content was gently extruded to confirm patency and induce polymicrobial peritonitis. The cecum was returned to the abdominal cavity, and the muscle and skin layers were closed sequentially. For fluid resuscitation, mice were immediately injected subcutaneously with warm sterile PBS (50 mL/kg). Sham-operated mice underwent an identical laparotomy and cecal exposure without the ligation and puncture procedures. CLP surgery was followed by close clinical monitoring (activity, body temperature, respiration, piloerection/dehydration), and predefined humane endpoints; mice showing persistent hypothermia, severe lethargy with inability to eat/drink, respiratory distress, or weight loss exceeding a preset threshold were euthanized immediately. Identical drug treatments were administered, and the mice were euthanized 24 h post-surgery for pathological and biochemical evaluations.

#### Wet-to-dry lung weight ratio (W/D ratio)

To evaluate the pulmonary edema, the lung wet-to-dry (W/D) weight ratio was determined. Following euthanasia, the left lungs were harvested and immediately weighed to obtain the wet weight. Subsequently, the lung tissues were placed in an oven at 80°C for 48 h to achieve the dry weight. The lung W/D weight ratio was calculated by dividing the wet weight by the dry weight.

#### Histological analyses of lung tissue

Histological analyses were performed to evaluate lung tissue injury and inflammation. Briefly, the left upper lobe of each lung was collected, fixed in 4% paraformaldehyde, paraffin-embedded, sectioned, and stained with H&E. Histopathological lung injury was scored in a blinded manner according to the American Thoracic Society (ATS)-recommended scoring system.^36^ For every mouse, 20 random, non-overlapping fields were examined. Five parameters were assessed in each field: (A) neutrophils in the alveolar space, (B) neutrophils in the interstitial space, (C) hyaline membranes, (D) proteinaceous debris filling the airspaces, and (E) alveolar septal thickening. Each parameter was scored as 0, 1, or 2 according to severity: for alveolar or interstitial neutrophils, 0 = none, 1 = 1–5 cells, and 2 = >5 cells; for hyaline membranes, 0 = none, 1 = one membrane, and 2 = more than one membrane; for proteinaceous debris, 0 = none, 1 = one instance, and 2 = more than one instance; for alveolar septal thickening, 0 = less than two times the thickness of sham/control alveolar septa, 1 = two-to 4-fold thickening, and 2 = more than 4-fold thickening. The lung injury score for each field was calculated as: [(20 × A) + (14 × B) + (7 × C) + (7 × D) + (2 × E)]/100. The final lung injury score for each mouse was obtained by averaging the scores from all analyzed fields.

Regarding the local inflammation of lung tissue, the immunofluorescence staining was utilized to measure the expression of pro-inflammatory cytokines and activation of pro-inflammatory cells. The primary antibodies against IL-1β (Abmart, TA5103, 1:200), IL-6 (Abmart, TD6087, 1: 200), TNFα (Abmart, PY19810S, 1:500), F4/80 (Abcam, ab300421, 1:100), LY6G (Proteintech, 65078-1-Ig, 1:100) and CCR7 (Proteintech, 25898-1-AP, 1:200) were utilized in the immunofluorescence analyses.

#### Western Blot analyses of lung tissue

The activation of classical inflammatory pathways, namely TLR4-MYD88-NF-κB and MAPK/ERK/JNK pathways, of lung tissue was measured by Western Blot (WB). Lung tissues were homogenized in RIPA Lysis Buffer (Beyotime, P0013B) containing protease and phosphatase inhibitors. Lysates were centrifuged at 12000 rpm for 20 min, and the supernatant protein concentration was determined using a BCA kit (Beyotime, P0012). Equal amounts of protein (30 μg per lane) were separated by SDS–PAGE and transferred to PVDF membranes. Membranes were blocked with 5% non-fat milk for 1 h at room temperature and incubated with primary antibodies overnight at 4°C, followed by incubation with appropriate HRP-conjugated secondary antibodies for 1 h at room temperature. Bands were visualized using ECL substrate (Beyotime, P0018S). Target signals were normalized to β-tubulin. The following antibodies were used: TLR4: Proteintech, 19811-1-AP, 1:500; MYD88: Servicebio, GB111554, 1:1000; NF-κB: Servicebio, GB153882, 1:1000; p38 MAPK: CST, 8690T, 1:1000; ERK: Servicebio, GB11560, 1:1000; *p*-ERK: Servicebio, GB11004, 1:1000; JNK: Servicebio, GB114321, 1:1000; *p*-JNK: Servicebio, GB12018, 1:1000; β-Tubulin:Proteintech, 10094-1-AP, 1:10000. To further evaluate the M2 polarization of macrophage and its upstream regulatory mechanisms, the protein levels of iNOS, PPAR-γ, and Arg-1 in lung tissues were also examined. The preparation and processing procedures were identical to those described previously. The Western blot bands with molecular weight markers are shown in Figure S17. The antibodies used are listed as follows: iNOS: Proteintech, 18985-1-AP, 1:1000; PPAR-γ: Proteintech, 66936-1-Ig, 1:10000; Arginase-1 (Arg-1): Proteintech, 16001-1-AP, 1:30000. Four biologically independent mice were analyzed per group (*n* = 4).

#### Blood routine tests

One day after the injection, the anticoagulant blood was taken from the heart for further blood routine tests, which was provided by the Servicebio company (https://www.servicebio.cn/). Five mice were included in each group.

#### Flow cytometry of BALF

The BALF collection and the flow cytometry analysis were performed according to our previous study.^35^ The immune cells were acquired by centrifugation at 350*g* for 5 min. After being washed by PBS, the cells were resuspended in 50 μL buffer for the stain of markers of Live/Dead (Thermo, L34955) and the activation of immune cells as following.

The Data acquisition was performed on a BD FACSCanto II flow cytometer, and the data analysis was performed by the FlowJo software (RRID:SCR_008520). After the doublets and dead cells being excluded, neutrophils were identified as CD45^+^ CD11b^+^ Ly6G^+^ cells, and macrophages were identified as CD45^+^ CD11b^+^ Ly6G^−^ cells. Macrophage polarization was assessed by the mean fluorescence intensity (MFI) of CD86 and CD206.

#### Protein concentration of BALF

The BALF was centrifuged at the speed of 2500 r/min for 10 min, and the supernatant was obtained for further analysis. The protein concentration of BALF was determined by BCA (Beyotime, P0012) according to the instructions. Four mice were included in each group.

#### Multiplex ELISA of lung and serum

500 μL whole blood was taken of each mouse and the serum was acquired by centrifugation (5000 rpm, 10 min). The concentration of total protein of lung tissue was assessed by BCA protein assay kit. Further, the obtained protein was used for multiplex ELISA to evaluate the local and global inflammation (Boster Bro, MEK1003, 13-plex). Three mice were included in each group.

#### RNA sequencing of lung tissue

Total RNA was extracted from the left lower lobe using TRIzol reagent (Thermo, 15596018CN). Libraries were prepared using a poly(A)-enrichment mRNA library preparation workflow and sequenced on an Illumina platform to generate paired-end reads. After quality control and adaptor trimming, reads were aligned to the mouse reference genome, and gene-level counts were generated for differential expression analysis. Differential expression analysis was performed using DESeq2 (RRID:SCR_015687), with FDR-adjusted *p* < 0.05 and |log_2_FC| > 1 as thresholds for differentially expressed genes (DEGs).^37^ GO and GSEA enrichment analyses were conducted to explore the functional and biological implications of the identified DEGs. Three mice were included in each group.

#### Sample allocation statement

Five mice per group were initially included in the *in vivo* study. For primary phenotypic assessments, including body weight change, lung wet-to-dry ratio, histological scoring and routine blood tests, all mice were analyzed. For downstream assays, the number of biological replicates varied depending on prespecified sample allocation, tissue availability, and assay throughput. Specifically, flow cytometry, immunofluorescence, and western blot analyses were performed with four biologically independent samples per group, whereas multiplex cytokine ELISA and RNA-seq were performed with three biologically independent samples per group. For assays performed on a subset of samples, samples were selected based on prespecified assay allocation and sample availability, not on the basis of experimental outcome.

### Quantification and statistical analysis

All statistical analyses were performed using SPSS (IBM). The exact value of n, the definition of the experimental unit, and the statistical tests used for each experiment are indicated in the corresponding figure legends. For animal experiments, n denotes the number of biologically independent mice per group. For cell-based assays, n denotes independent biological experiments performed on separate occasions. For characterization and particle-tracking assays, n is defined in the relevant figure legends. Data are presented as mean ± standard deviation (SD). Comparisons among multiple groups were performed using ordinary one-way analysis of variance (ANOVA) followed by Tukey’s post-hoc test for multiple comparisons. *p* < 0.05 was considered statistically significant. (∗*p* < 0.05, ∗∗*p* < 0.01, ∗∗∗*p* < 0.001). The exact statistic value of ANOVA analysis and the Tukey’s post-hoc test was also provided in the Supplemental Item (Data S1).

## Supplemental information

Document S1. Figures S1–S17 and Table S1

Data S1. The exact statistic value of ANOVA analysis and the Tukey’s post hoc test, related to Figures 3, 4, 5, and 6
