# Supplementary material for: Tetracycline-loaded nanomotors target neutrophil activation and oxidative stress in acute lung injury
Source: iScience. 2026 Aug 6;29(8):117106. doi: 10.1016/j.isci.2026.117106 (PMC13475673; doi:10.1016/j.isci.2026.117106)
Supplement: Supplementary file 1 [file mmc1.pdf]

iScience, Volume *29*

Supplemental information

Tetracycline-loaded nanomotors target neutrophil activation and oxidative stress in acute lung injury

Saiqi Li, Ziqiang Zhang, Qianyun Zhang, Yi Shen, Chong Zhang, Jia Huang, Fan Su, and Yuning Sun

Supporting Information

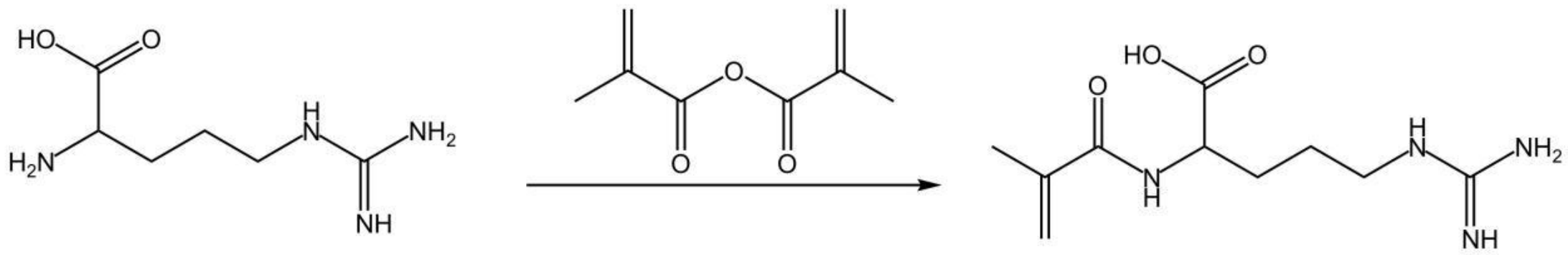

Figure S1. Synthesis of MA Monomer.

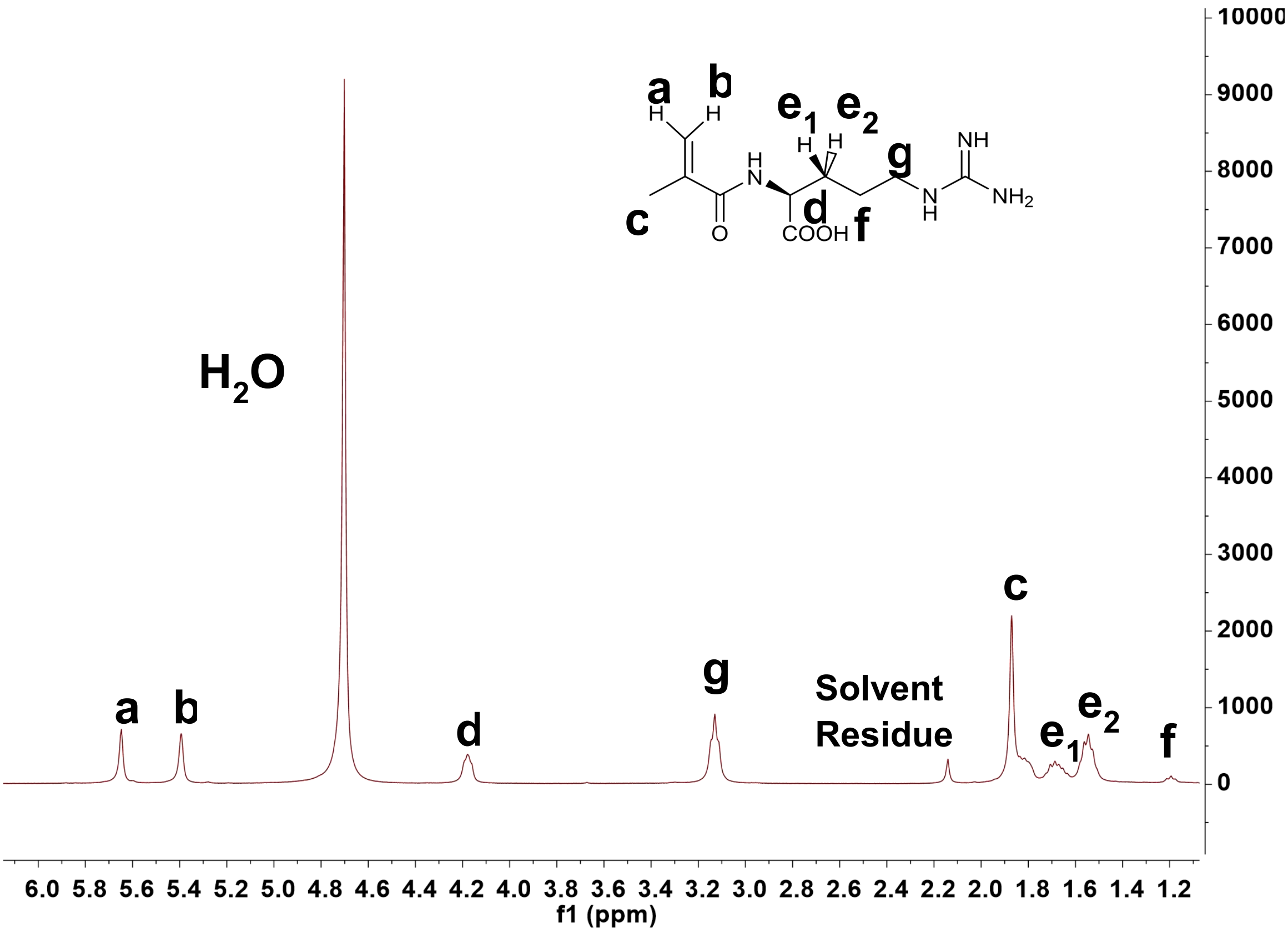

Figure S2. ^{1}H-NMR Spectrum of MA**.

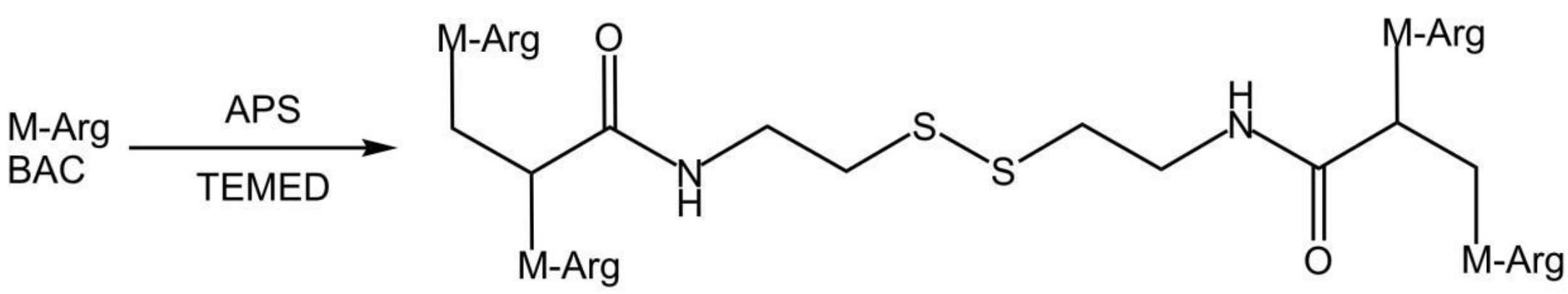

Figure S3. Synthesis of PMA Nanomotors.

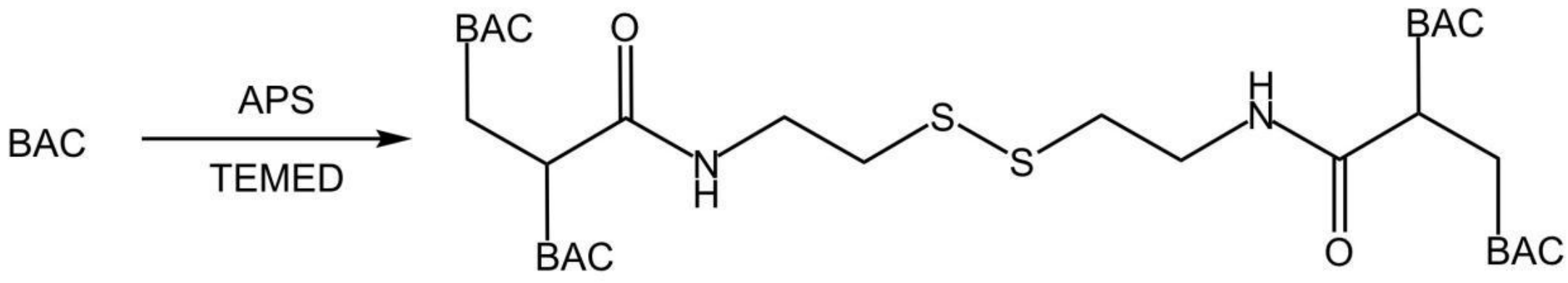

Figure S4. Synthesis of Control PB.

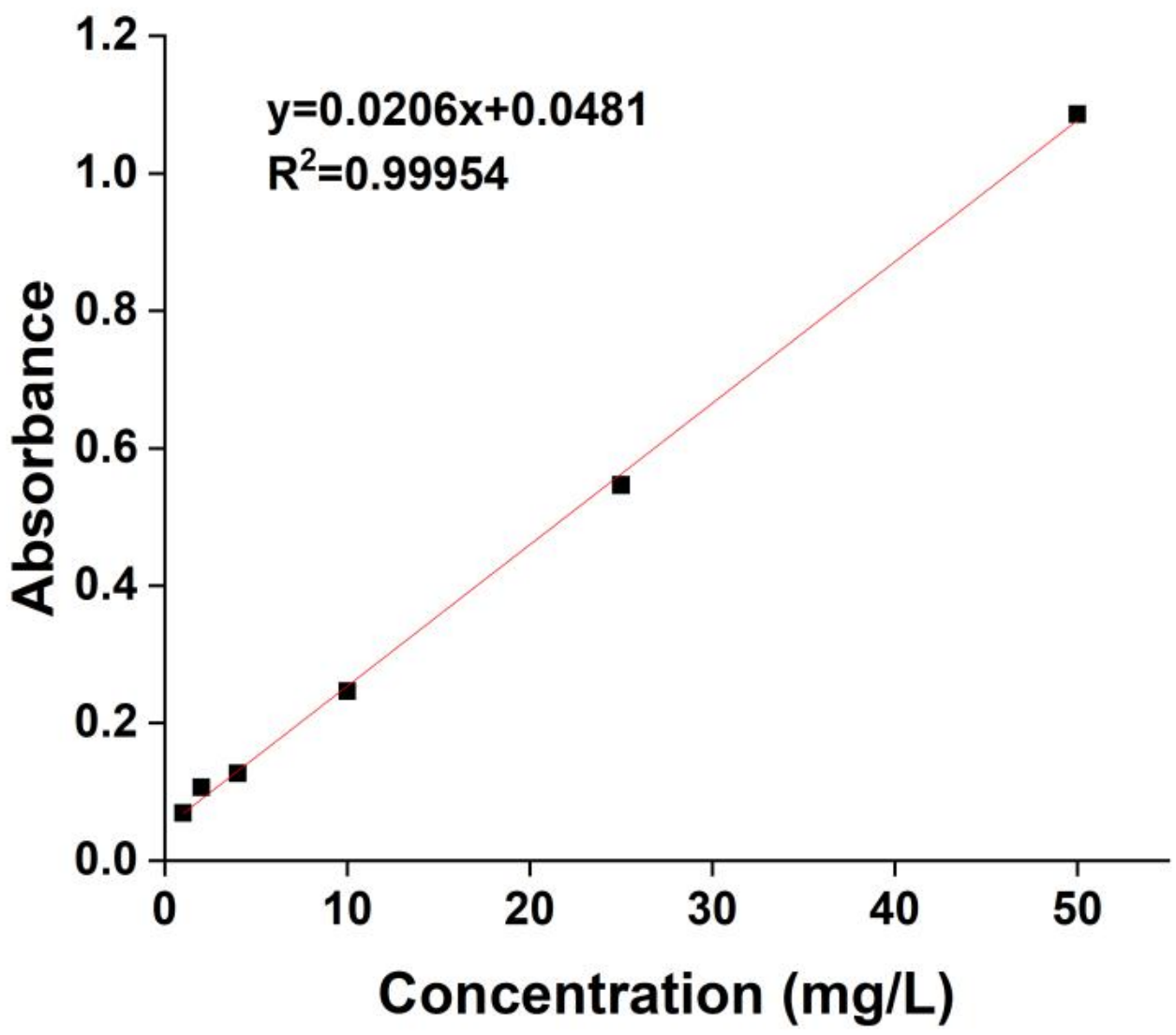

Figure S5. TH Standard Curve.

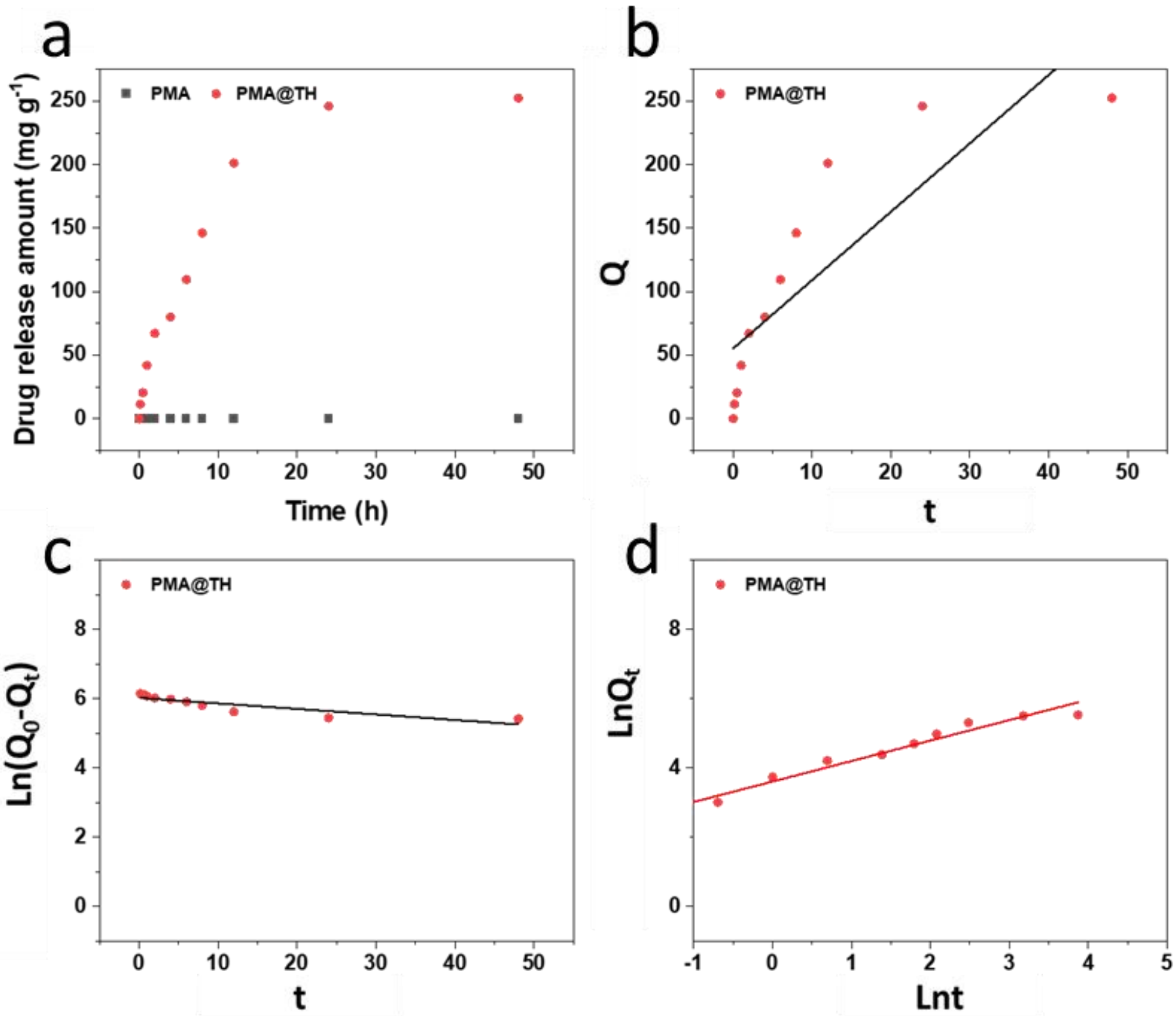

Figure S6. The fitting of drug release curves under different models: (a) drug release profile, (b) Zero-order model, (c) First-order model, (d) Peppas model.

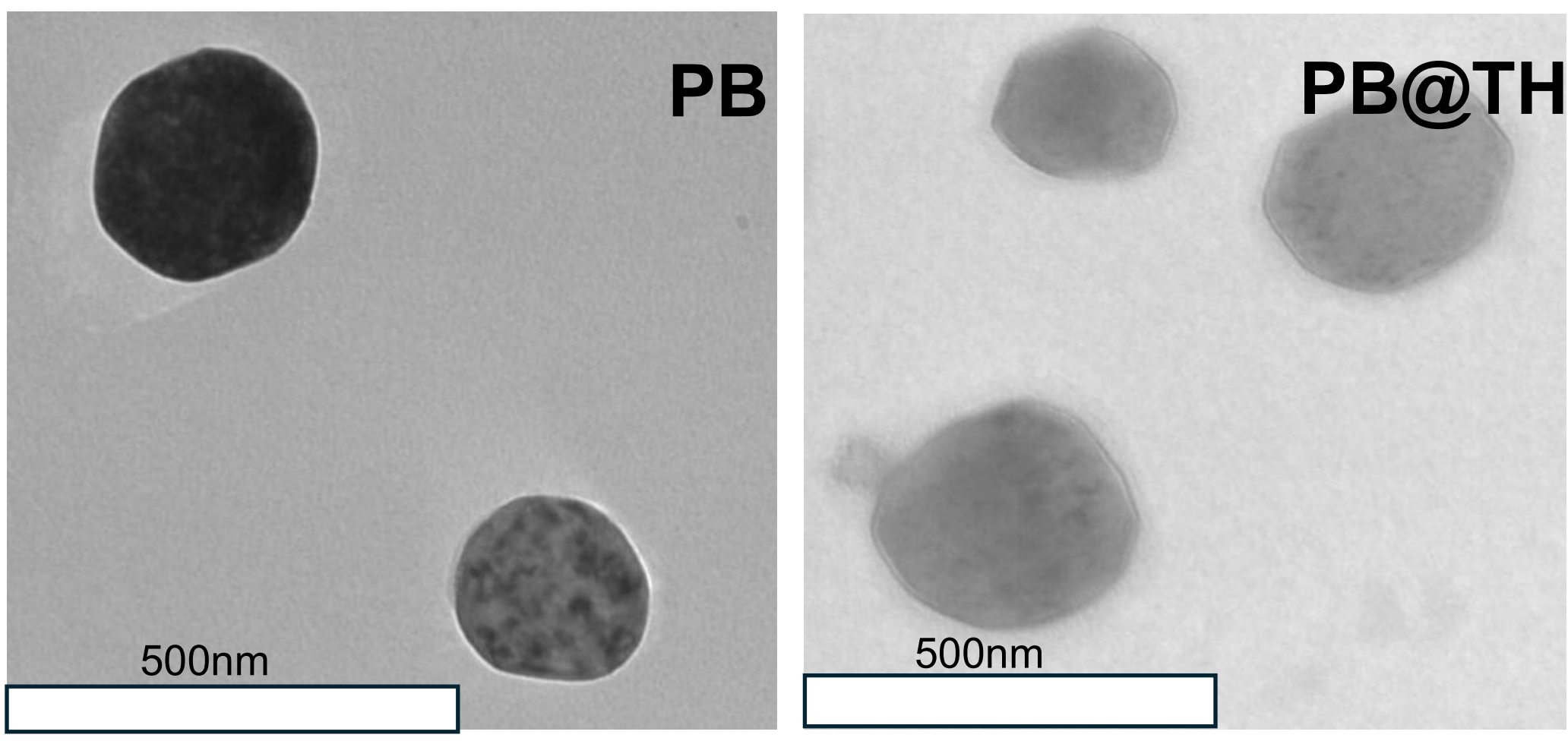

Figure S7. TEM Images of PB and PB@TH, scale bar = 500 nm.

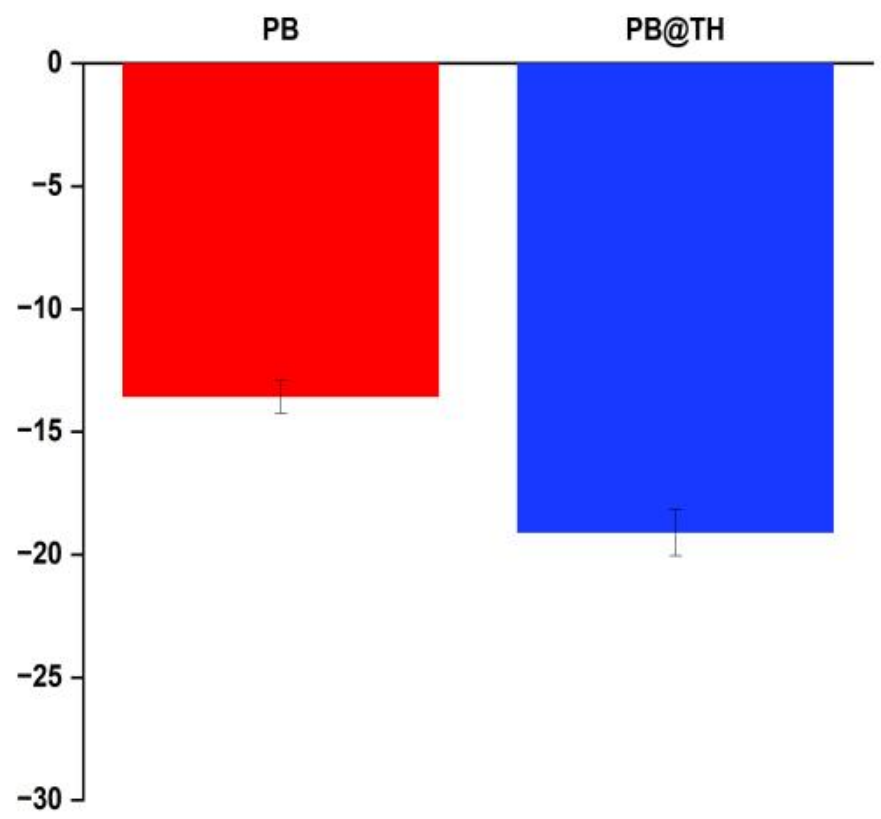

Figure S8. Zeta Potential of PB and PB@TH. Data are shown as mean ± SD.

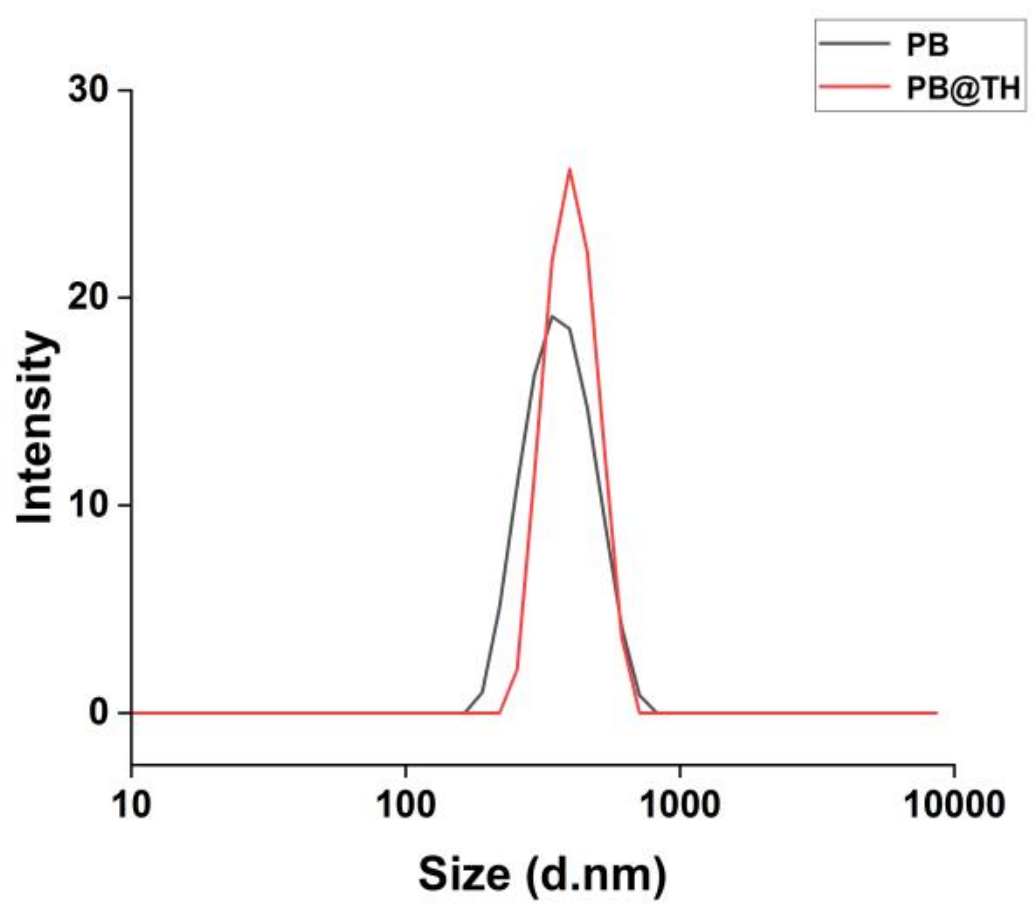

Figure S9. Size Distribution of PB and PB@TH.

a- PMA@TH

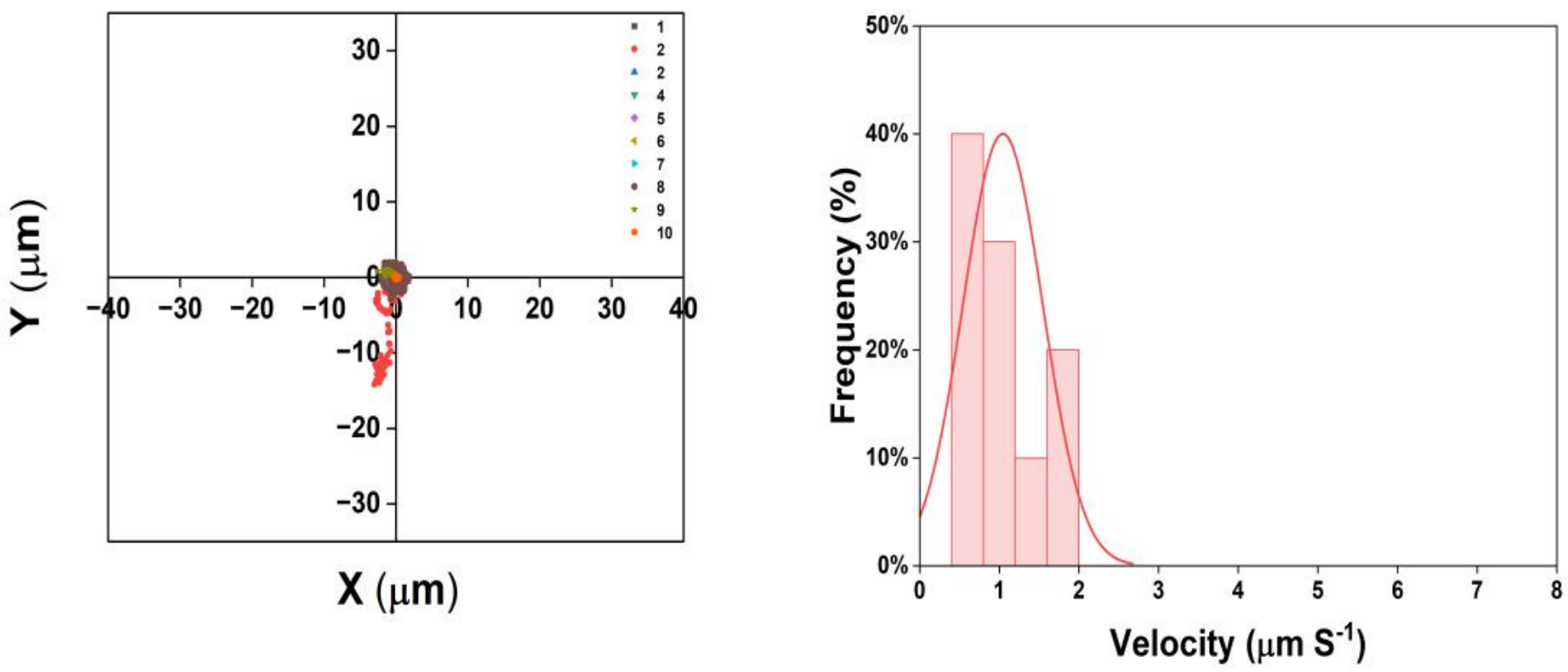

b- PB@TH-LPS

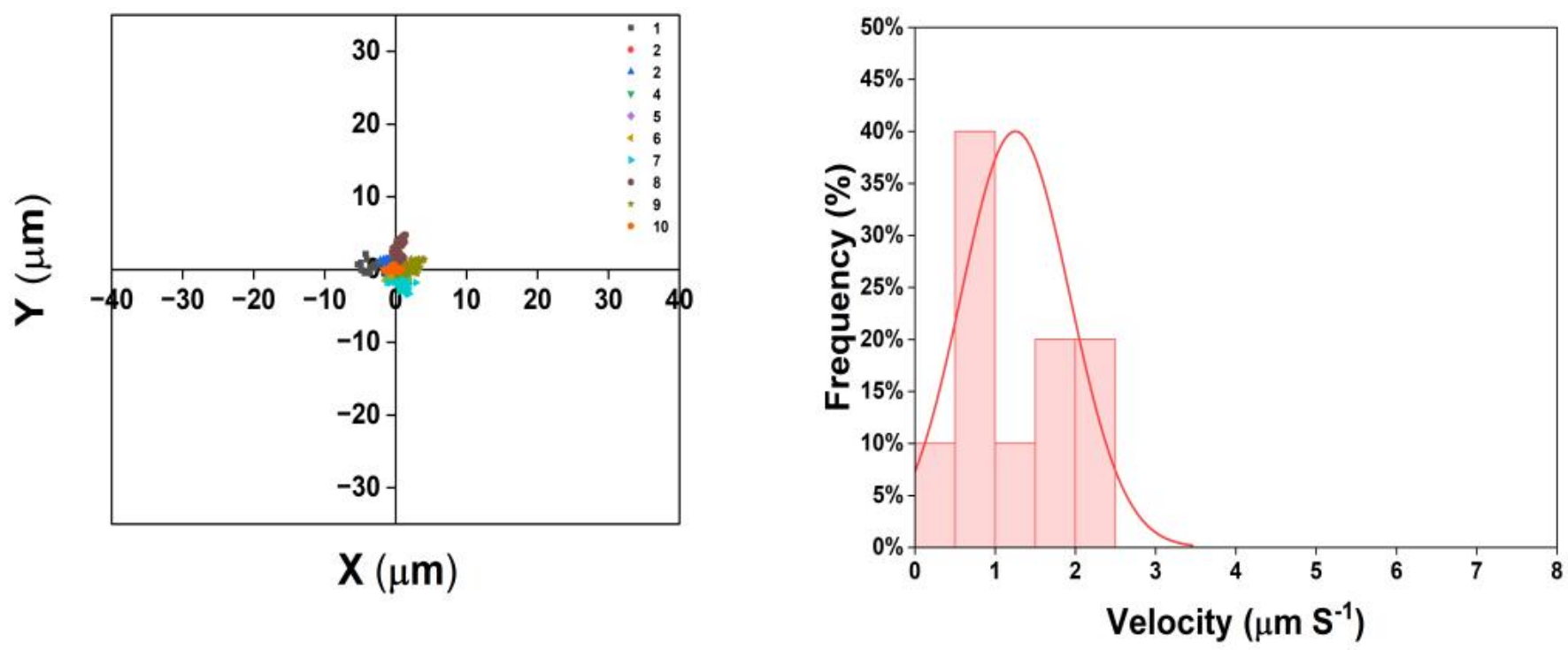

c- PB@TH

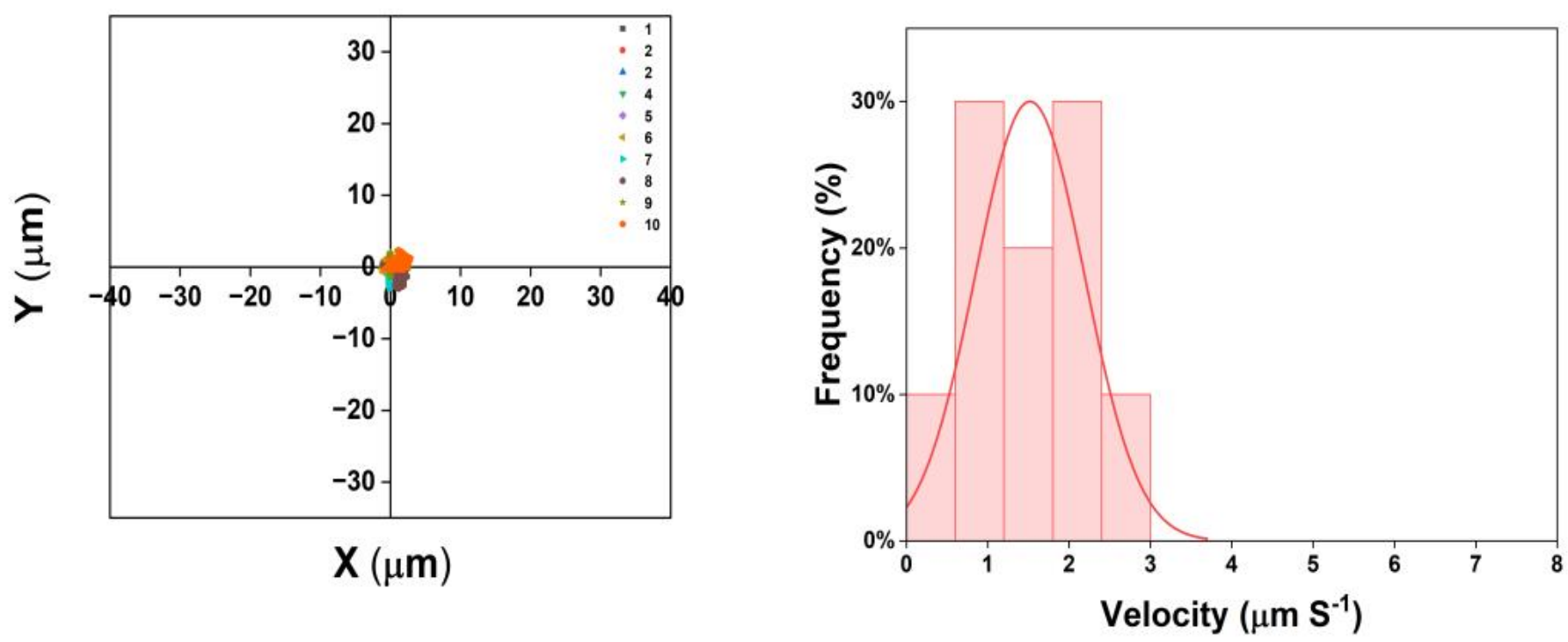

Figure S10. Motion of PMA@TH in raw macrophages, PB@TH in LPS-Stimulated (b) and raw macrophages (c).

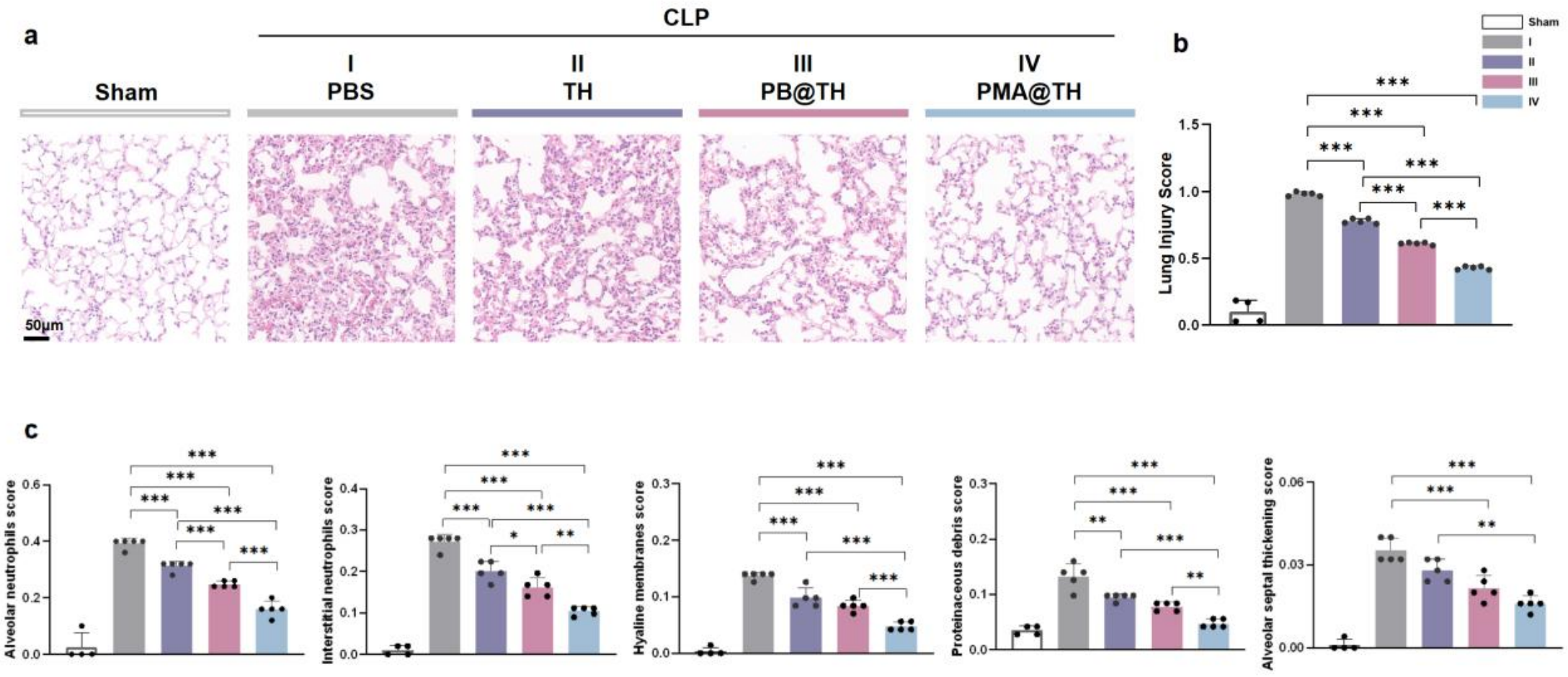

Figure S11. MA@TH inhibits inflammatory lung injury in CLP- induced ALI mice. (a) The representative images of H&E-stained lung sections in CLP mice (scale bar, 50 μm). (b) The total lung injury score based on the histological images. (c) The five independent features of the lung injury score (n = 3 technical replicates from 5 biological replicates for each strain). Statistical analysis: One-way ANOVA followed by Tukey's multiple comparisons test. Data are shown as mean ± SD. *P < 0.05, **P < 0.01, ***P < 0.001. Groups: I, ALI; II, ALI + TH; III, ALI + PB@TH; IV, ALI + PMA@TH.

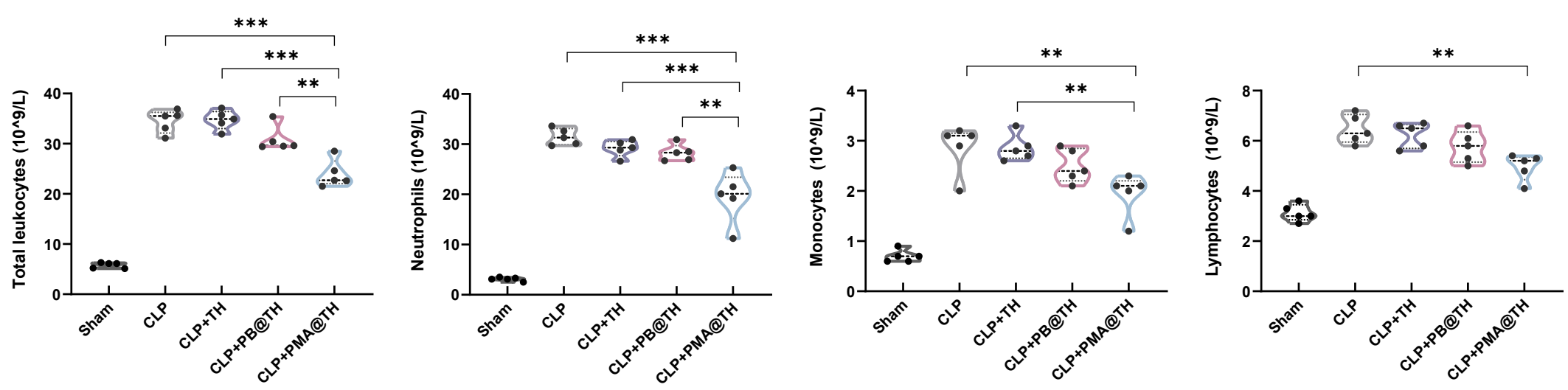

Figure S12. MA@TH reduced the counts of white blood cells in the blood of CLP mice (n = 3 technical replicates from 5 biological replicates for each strain). Statistical analysis: One-way ANOVA followed by Tukey's multiple comparisons test. Data are shown as mean ± SD. *P < 0.05, **P < 0.01, ***P < 0.001.

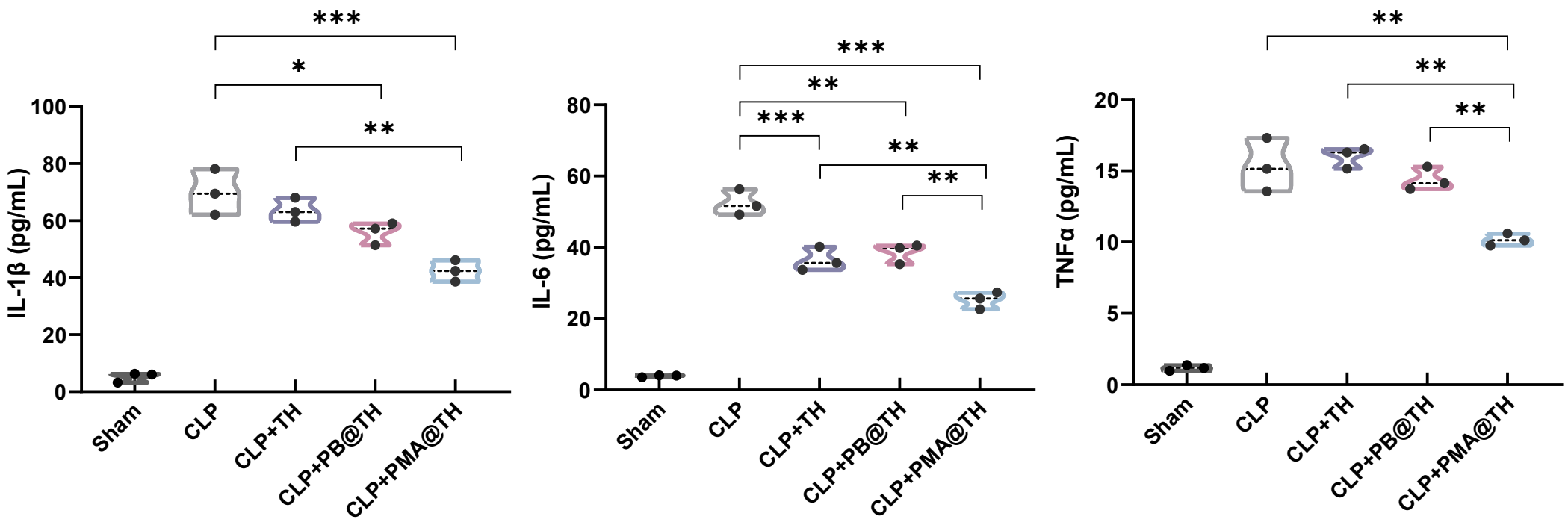

Figure S13. MA@TH reduced the levels of pro- inflammatory cytokines in the serum of CLP mice (n = 3 technical replicates from 3 biological replicates for each strain). Statistical analysis: One-way ANOVA followed by Tukey's multiple comparisons test. Data are shown as mean ± SD. *P < 0.05, **P < 0.01, ***P < 0.001.

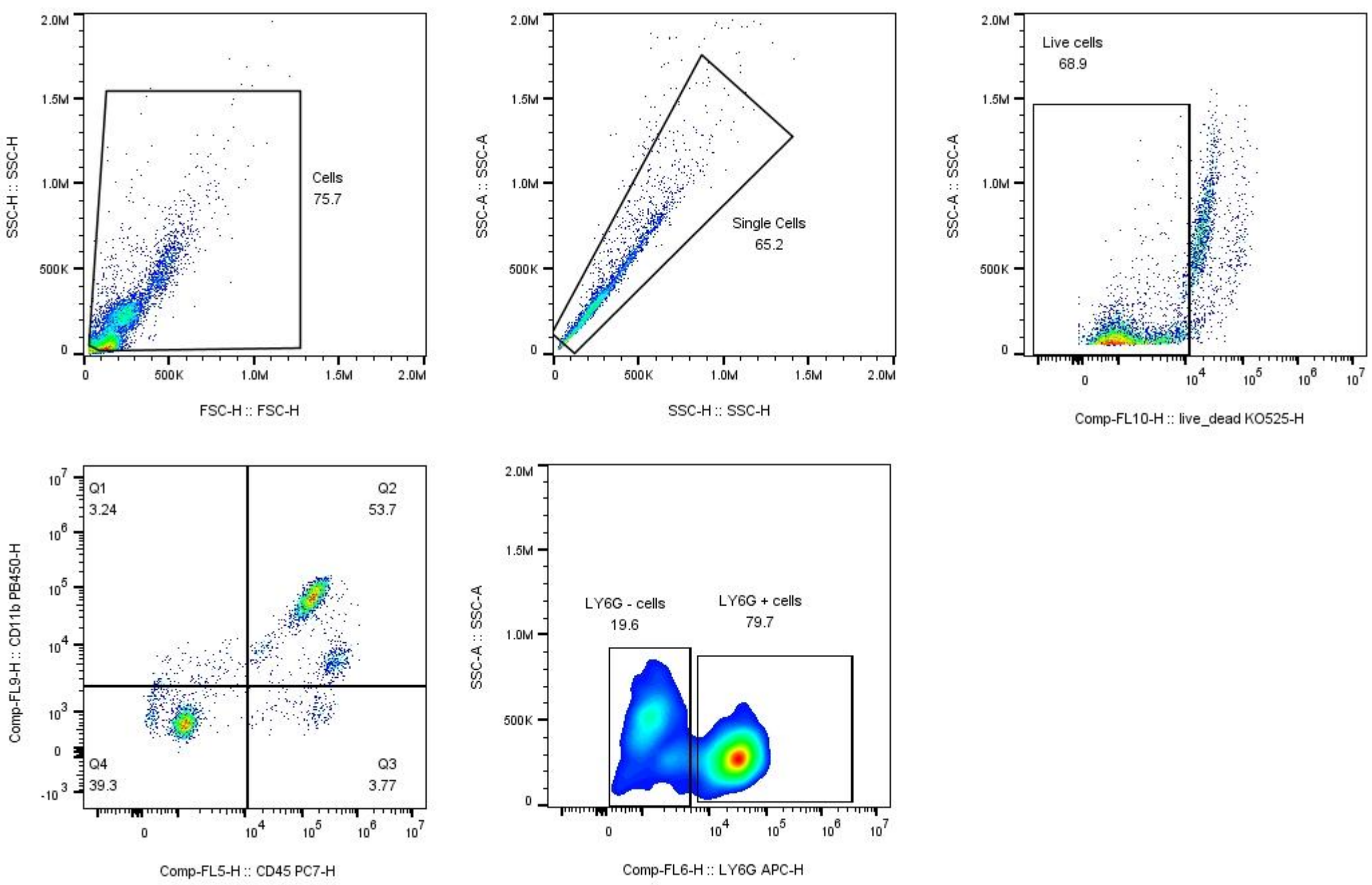

Figure S14. The gating strategy of the inflammatory cells in the BALF.

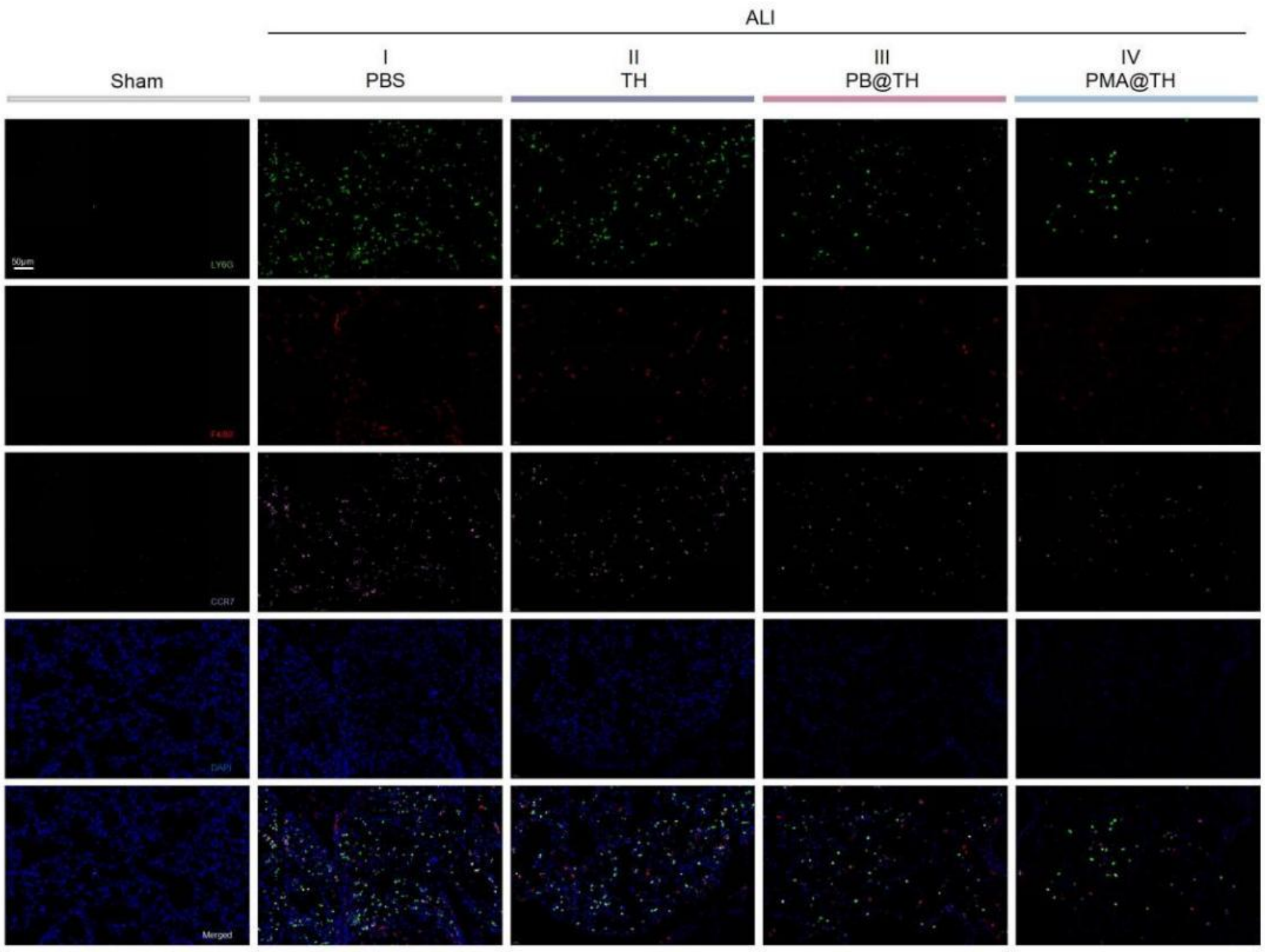

Figure S15. The infiltration of inflammatory cells in the lung. The expressions of LY6G, F4/80 and CCR7 were significantly reduced by PH, PB@TH and PMA@TH (scale bar, 50 μm). PMA@TH displayed the the most potent inhibition effects.

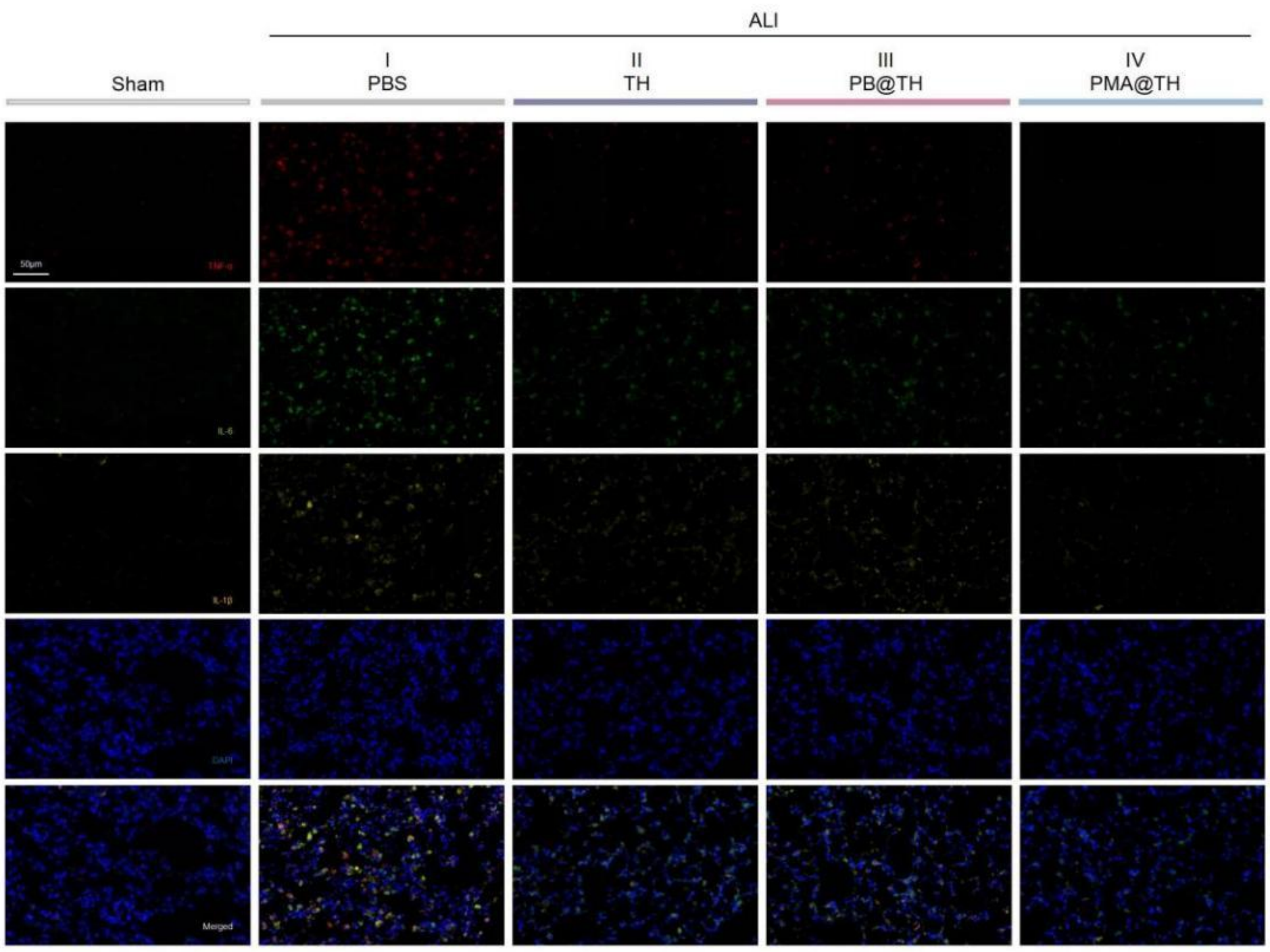

Figure S16. The expression of cytokine in the lung. The expressions of TNFα, IL-6 and IL-1β were significantly reduced by PH, PB@TH and PMA@TH (scale bar, 50 μm). PMA@TH displayed the the most potent inhibition effects.

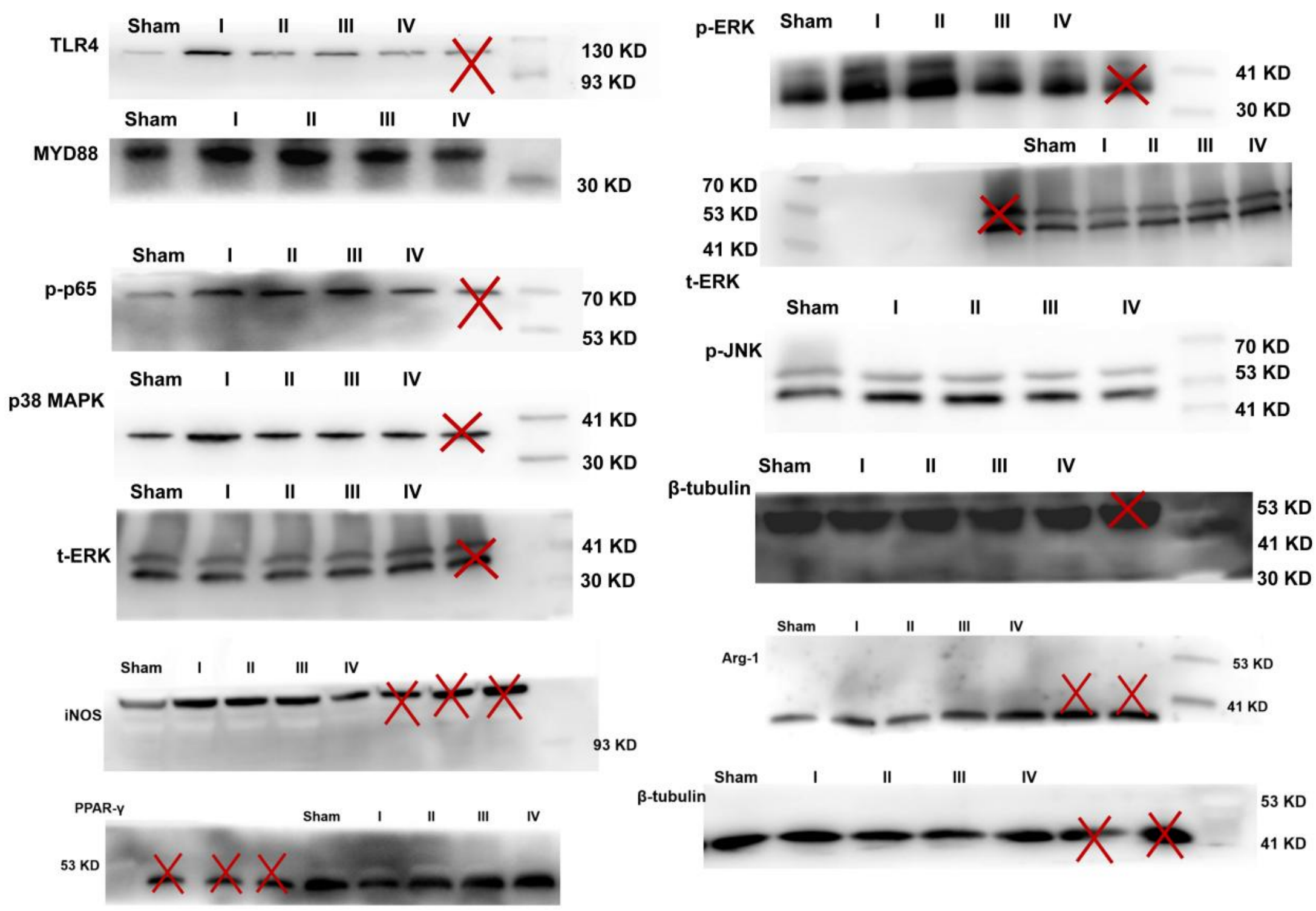

Figure S17. The WB blots accompanied by the locations of molecular markers.

Table S1. Fitting parameters under different drug release models.

Model	R^2	k	lna	b
Zero-order (0-48 h)	0.6914	5.3862±1.1133	---	---
First-order (0-48 h)	0.7534	0.0166±0.0030	---	---
Peppas (0-48h)	0.9648	---	3.6095±0.079	0.5908±-0.037
